# A No-History Multi-Formula Approach to Improve the IOL Power Calculation after Laser Refractive Surgery: Preliminary Results

**DOI:** 10.3390/jcm12082890

**Published:** 2023-04-15

**Authors:** Ferdinando Cione, Maddalena De Bernardo, Marco Gioia, Marianunzia Oliviero, Andrea Giuseppe Santoro, Alessandro Caputo, Luigi Capasso, Sergio Pagliarulo, Nicola Rosa

**Affiliations:** 1 Eye Unit, Department of Medicine Surgery and Dentistry, Scuola Medica Salernitana, University of Salerno, 84081 Baronissi, Italy; fcione@unisa.it (F.C.); mgioia@unisa.it (M.G.); nrosa@unisa.it (N.R.); 2 Corneal Transplant Unit, ASL Napoli 1, 80100 Naples, Italy

**Keywords:** cataract surgery, refractive surgery, IOL power calculation, multi-formula approach

## Abstract

This retrospective comparative study proposes a multi-formula approach by comparing no-history IOL power calculation methods after myopic laser-refractive-surgery (LRS). One-hundred-thirty-two eyes of 132 patients who had myopic-LRS and cataract surgery were examined. ALMA, Barrett True-K (TK), Ferrara, Jin, Kim, Latkany and Shammas methods were evaluated in order to back-calculate refractive prediction error (PE). To eliminate any systematic error, constant optimization through zeroing-out the mean error (ME) was performed for each formula. Median absolute error (MedAE) and percentage of eyes within ±0.50 and ±1.00 diopters (D) of PE were analyzed. PEs were plotted with corresponding mean keratometry (K), axial length (AL), and AL/K ratio; then, different ranges were evaluated. With optimized constants through zeroing-out ME (90 eyes), ALMA was better when K ≤ 38.00 D-AL > 28.00 mm and when 38.00 D < K ≤ 40.00 D-26.50 mm < AL ≤ 29.50 mm; Barrett-TK was better when K ≤ 38.00 D-AL ≤ 26.50 mm and when K > 40.00 D-AL ≤ 28.00 mm or AL > 29.50 mm; and both ALMA and Barrett-TK were better in other ranges. (*p* < 0.05) Without modified constants (132 eyes), ALMA was better when K > 38.00 D-AL ≤ 29.50 mm and when 36.00 < K ≤ 38.00 D-AL ≤ 26.50 mm; Barrett-TK was better when K ≤ 36.00 D and when K ≤ 38.00 D with AL > 29.50 mm; and both ALMA and Barrett-TK were better in other ranges (*p* < 0.05). A multi-formula approach, according to different ranges of K and AL, could improve refractive outcomes in post-myopic-LRS eyes.

## 1. Introduction

Due to the excellent results achieved, myopic laser refractive surgery (LRS), such as photorefractive keratectomy (PRK), laser in situ keratomileusis (LASIK), and laser-assisted subepithelial keratectomy (LASEK), has been widely used in recent decades [1,2].

It is well known that LRS can induce changes that involve not only the cornea, but also other ocular structures [3]. These changes can make several measurements, such as the intraocular pressure and the corneal power, unreliable [4,5,6,7,8,9].

For these reasons, intraocular lens (IOL) power calculation will be challenging in these patients [10]. After myopic-LRS, most devices measure a steeper anterior corneal curvature as they extrapolate the central corneal curvature from paracentral measurements. This event, in addition to a change in the keratometric index, causes incorrect estimation of the effective lens position (ELP), as all these parameters are mandatory for an accurate calculation [11,12]. In fact, after cataract surgery in post-myopic-LRS eyes utilizing most of the routinely used formulas, the so-called “hyperopic surprise” is common [13].

Most of the methods proposed to overcome this problem require the knowledge of pre-refractive surgery data, such as the amount of treatment, but, disappointingly, this information is not available for most patients [14]. For this reason, new methods that did not require the knowledge of pre-refractive surgery data were proposed. These formulas are also known as “no-history” methods because they do not require historical data, such as the amount of refractive treatment or preoperative refractive error.

Recently, several studies compared different formulas trying to establish which could be the best in all cases, though only four analyzed no-history formulas in eyes with prior myopic-LRS according to their axial length (AL), but none attempted to check if different methods could deliver better results if utilized in patients with different mean keratometry (K) or AL/K ratio [15,16,17,18,19].

The aim of this study was to calculate and analyze differences in the refractive prediction errors (PE) between different formulas looking at different K and AL ranges when using no-history methods in post myopic-LRS eyes.

This study was performed according to protocols published by Hoffer et al. [20], recently updated [21], and by Holladay et al. [22] A cornerstone of these protocols is represented by the mandatory need to optimize the lens constant: constant optimization is the process-leading mean PEs (ME) of each analyzed sample equal to zero (zeroing-out) in order to eliminate any systematic error [21]. In fact, if the ME is different from zero, an inappropriate lens factor for that specific group is used [20]. Since some papers suggest that zeroing-out the ME should not be done for atypical eyes [23], this study, similarly to others [16,17,18,19,20,21,22,23,24], was performed both with and without zeroing-out the ME.

## 2. Materials and Methods

In this multicenter retrospective study, patients who underwent uneventful cataract extraction and IOL implantation in the capsular bag after myopic LASIK or PRK were evaluated.

### 2.1. Inclusion and Exclusion Criteria

The research followed the tenets of the Declaration of Helsinki and preoperative informed consent from the patients was obtained before cataract surgery, including consent to use anonymized medical information for scientific purposes [25].

Due to the multicenter nature of the study, patients were recruited in several eye units of hospitals and clinics in Italy, but final evaluation of postoperative data was performed at Eye Unit, University of Salerno, the data collection center. In addition, surgeons of satellite centers provided preoperative and postoperative data to the collection center. Institutional Review Board (IRB) approval to perform such analysis was obtained. In addition, a part of patients’ database was taken from the international literature: only eyes in which biometric and refractive data were available were chosen, applying the same inclusion and exclusion criteria used for other eyes [25,26]. Literature data came from the Dutch Association for Refractive Surgeon [26] or Kangnam St. Mary’s Hospital, Seoul [25].

A total of 446 eyes that underwent cataract surgery with previous myopic LASIK or PRK surgery from January 2014 to December 2021 were eligible for the study. Patients without previous refractive surgery or that underwent refractive surgery different from myopic LASIK or PRK (e.g., hyperopic LASIK) were not considered eligible for the study.

From the total of eyes considered eligible for the study, 314 eyes were excluded after applying exclusion criteria:

- 24 eyes because keratometric readings were not obtained with keratometry:
- 239 eyes because not all postoperative data were known (unknown refraction after cataract extraction or unknown implanted IOL power, this large loss of patients’ data is due to the multicenter nature of the study);
- 34 eyes because of confounding retinal or corneal diseases (severe dry eye, pterygium, eye surface diseases);
- 17 eyes because of complications associated with cataract surgery or because they had a postoperative corrected distance visual acuity ≤20/40 [21].

After applying the inclusion and exclusion criteria, a total of 132 eyes (Group A) were selected for analysis. In all subjects, cataract surgeries were performed by different surgeons in different clinics by standard phacoemulsification with all IOLs placed in the capsular bag.

In all eyes, the following data were available: K and AL prior to phacoemulsification, A-constant, power of the implanted IOL, and refractive outcome at least one month after surgery.

Selected data were not previously used to develop any of the formulas under examination [21].

K readings and AL measurements were obtained with partial coherence interferometry (PCI) performed with an IOLMaster 500 (Software version 5.4.4.0006; Carl Zeiss Meditec, Jena, Germany,). Patients’ spherical equivalent postoperative refractions were obtained through subjective methods (performed by the surgeon) after an autorefractometer evaluation. For IOL power calculation methods other than the SRK/T [27] based formulas, A-constants were converted using standard relations.

### 2.2. Analyzed IOL Power Formulas

Among the methods that do not require the use of preoperative parameters or amount of treatment to calculate the IOL power [11,24,26,28,29,30,31,32,33,34,35,36,37,38,39,40,41], it was possible to evaluate the methods described by Rosa (ALMA) [24], Barrett (True-K) [30], Ferrara [31], Jin [34], Kim [26], Latkany (flat-K) [35], and Shammas [39].

Other no-history IOL power calculation methods after myopic-LRS were not analyzed because some of them require the use of special devices not available for analyzed patients [11,28,29,40]; others, such as the contact lens method [41] or the intraoperative refraction [33,36] because they required specific examinations that were not performed, and the Haigis-L no history formula [32] were not included because it requires the knowledge of the anterior chamber depth (ACD).

This parameter was recommended also for Barrett Universal II formula, the base of Barrett True-K (TK), together with other optional parameters, but it was demonstrated that the contribution of ACD was not clinically relevant in IOL power calculation when AL > 22.00 mm [42]. Lastly, the R factor method [38] was not analyzed because ALMA is its evolution.

### 2.3. IOL Power and Refractive Outcomes Calculation

To obtain the refractive outcomes, the following steps were performed utilizing Excel software (Microsoft Corporation, Redmond, WA, USA):

(1) Insertion of patients’ data in an Excel spreadsheet.

(2) Based on these data, predicted refractive outcomes were retrospectively calculated using seven different formulas.

ALMA [24], Ferrara [31], Jin [34], Kim [26], Latkany [35], and Shammas [39] methods were programmed into our database.

- Shammas clinically-derived K-value correction (Shammas c.d.) was used with Shammas-PL formula.
- Kim method was used with the SRK/T [27] formula.
- Jin method was used with the Holladay I [43] formula, aiming to −0.75 D.
- Ferrara, Latkany, and ALMA methods were used with SRK/T formula.
- Since Barrett-TK formula is still unpublished and the only way to calculate the IOL power with this method is through the American Society of Cataract and Refractive Surgery (ASCRS) Online Post-Refractive IOL Calculator (available at https://acrs.org/tools/iol-calculator, accessed on 24 February 2023) or the Asia Pacific Association of Cataract & Refractive Surgeon (APACRS) Online Post-Refractive IOL Calculator (available at https://www.apacrs.org, accessed on 24 February 2023), predicted refractive errors of Barrett-TK method were calculated through the APACRS IOL calculator.

(3) PE was calculated for each patient and for each formula. The difference between the post-operative refractive error and the predicted one gave the PE. The predicted refractive error was retrospectively calculated as the predicted refractive outcome with the implanted IOL by each method.

Among these patients, the zeroing-out of the ME was achievable in 90 eyes (Group B), because in some of them only the power and manufactory’s lens constant, but not the implanted IOL model, was known (25 eyes), or the IOL models were implanted in less than three patients (17 eyes), making the zeroing unreliable [24]. Group B was then composed only of eyes in which constant optimization through zeroing-out the ME was possible. No data from the literature were included in this group.

- To zero-out the ME with ALMA, Ferrara, Latkany, Kim, Jin, and Shammas methods, the obtained data were subdivided in groups according to the IOL models and the applied formulas. Then, the PEs were averaged and zeroed-out by applying the “goal seek” option for the “what f analysis” function in Excel [20,21].
- To zero-out the ME with the Barrett-TK method, a specific computer programming language was utilized (Python, Version 3.9.3, Python Software Foundation. Available at https://www.python.org, accessed on 14 August 2022). As reported in the previous paragraph, a Barrett-TK formula was used to calculate the predicted refraction for each patient, and it was then compared to the measured post-operative refraction and the ME for each group of patients was computed. This procedure was repeated while varying the A-constant and seeking to minimize the ME. The A-constant for which the ME was smallest (closest to 0.000 D) for each group of patients was noted.

The following values were calculated, in absolute values:

- Median absolute error (MedAE).
- Mean absolute error (MAE).
- Number and percentage of eyes within ±0.50 and ±1.00 D of PE.
- Minimum, maximum, and standard error, 95% confidence interval around the ME.
- Interquartile ranges (IQR) [21].

In both groups, PEs were plotted with the corresponding K and AL values, and they were divided in four ranges. Cut-off values of AL and K have been established in this way:

- AL: four ranges, based on the rounded to 0.50 mm AL value closer to each quartile value of Group B.
- K: four ranges, based on the rounded to 1.00 D K value closer to each quartile value of Group B.

Moreover, the parameter AL/K was identified. Each group was then divided into three subpopulations, based on the AL/K value closer to the fraction value of 2/3 (0.67) and ¾ (0.75).

### 2.4. Statistical Analysis

Descriptive statistics and statistical analysis were performed with SPSS software (Version 26.0 SPSS Inc., Chicago, IL, USA). The normality of data was examined by the exact Kolmogorov-Smirnov test before zeroing-out the ME.

For screening whether the ME was significantly different from zero, one-sample *t*-test was used.

For pair-wise comparison of absolute errors, the nonparametric Friedman’s test with Bonferroni correction was used. The Cochran Q test was used to compare the percentage of eyes within ±0.50 D and ±1.00 D of PE. A *p* value < 0.050 was considered statistically significant.

Required sample size was calculated with G*Power software (Version 3.1.9.7, Faul, Erdfelder, Lang, & Buchner, 2020. Available at https://www.gpower.hhu.de, accessed on 14 August 2022). Given a Partial η2 of 0.206, a non-sphericity correction ε of 0.357 corrected with Greenhouse-Geisser method, both calculated with SPSS software, and an effect size of 0.509. It was estimated that with a significance level of 5% and a test power of 85% [21], a sample size of 27 eyes would be necessary.

## 3. Results

All data were normally distributed (*p* > 0.050). All MEs were statistically different from zero (*p* < 0.050) except for the Shammas formula in Group B (*p* = 0.127), meaning that the lens factor chosen for this formula was more appropriate than others for this group of eyes [18].

Group A was composed by 132 eyes of 132 patients. AL measurements ranged from 23.72 to 34.20 mm, mean = 27.65 ± 2.11 mm, median = 27.44 mm, and K readings ranged from 31.56 to 43.81 D, mean = 38.08 ± 2.69 D, and median = 38.34 D.

Group B was composed by 90 eyes of 90 patients. In this group, AL measurements ranged from 23.72 to 34.20 mm, mean = 28.09 ± 2.13 mm, median = 27.97 mm, and K readings ranged from 31.56 to 43.17 D, mean = 37.64 ± 2.71 D, and median = 37.71 D.

The comparison among all IOL power calculation methods analyzed both in Groups A and B, Bonferroni post-hoc analysis and multiple comparisons according to the Cochran Q test are shown in Table 1 and in Supplementary Table S1.

**Group A**

**Figure 1:**
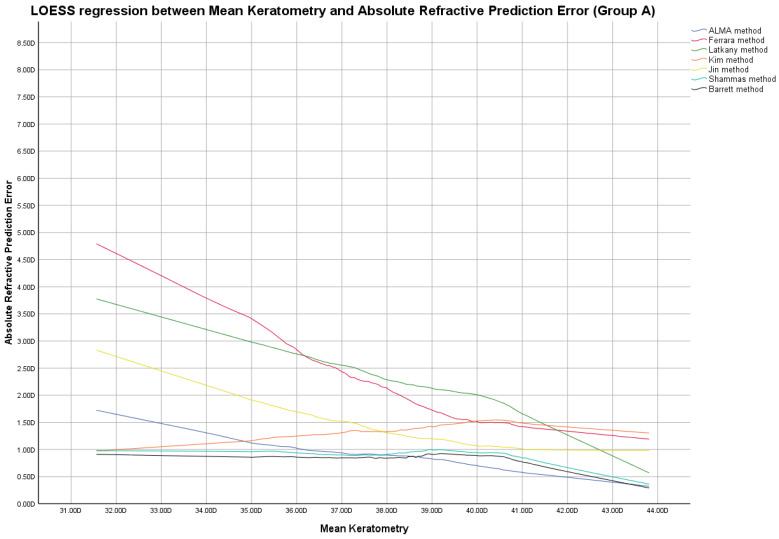
Locally estimated scatterplot smoothing (LOESS) curve between refractive prediction errors generated for each examined formula and the related mean keratometry value. (Group A).

**Figure 2:**
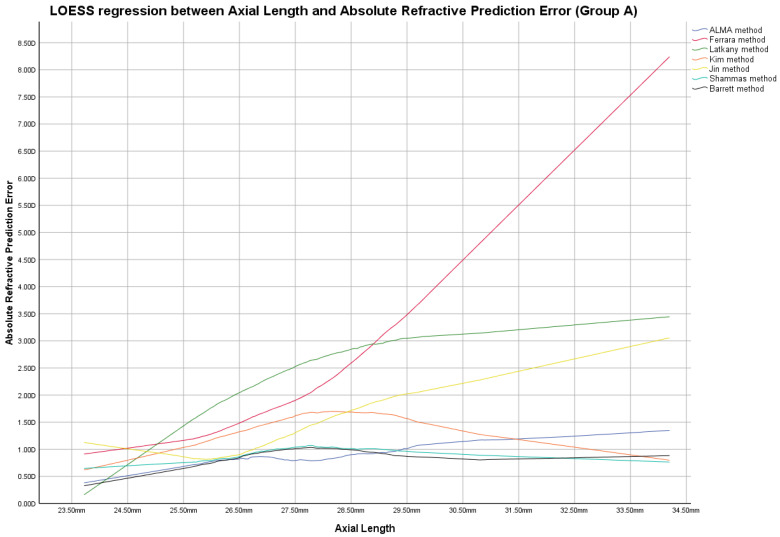
Locally estimated scatterplot smoothing (LOESS) curve between refractive prediction errors generated for each examined formula and the related axial length value. (Group A).

**Figure 3:**
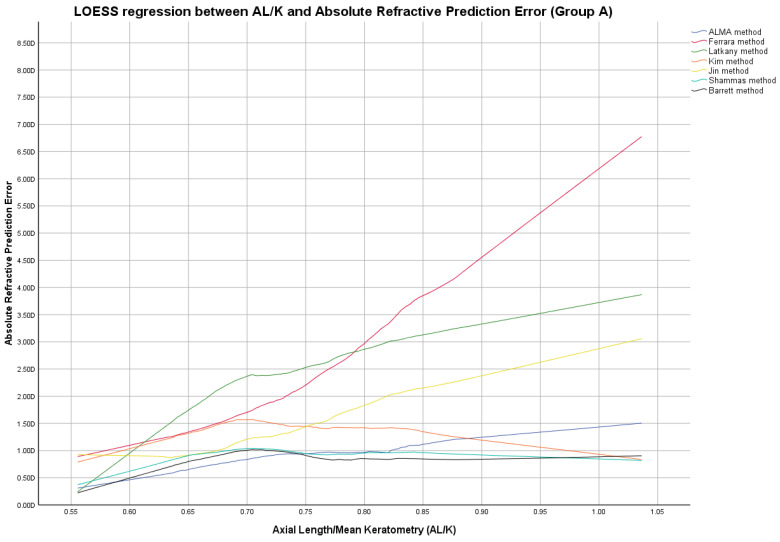
Locally estimated scatterplot smoothing (LOESS) curve between refractive prediction errors generated for each examined formula and the related axial length (AL)/mean keratometry (K) value. (Group A).

In this group, the ME was not zeroed-out and manufacturer’s suggested A-constants were utilized in the IOL power calculation, as shown in Supplementary Table S1. Figure 1, Figure 2 and Figure 3 report locally estimated scatterplot smoothing (LOESS) regression showing trend line of PEs according to K values, AL measurements, and AL/K ratio. The relationship among all IOL power calculation methods analyzed for all patients in Group A plotted with the K values, AL measurements, and AL/K ratio are shown in Table 2, Table 3 and Table 4 and Supplementary Table S2. Bonferroni post-hoc analysis and multiple comparisons according to the Cochran Q test for each analyzed range are shown in Supplementary Tables S3 and S4. Boxplot diagrams and bar graphs reporting refractive outcomes obtained in each individual analyzed range were reported in Supplementary Figures S1–S3.

**Group B**

**Figure 4:**
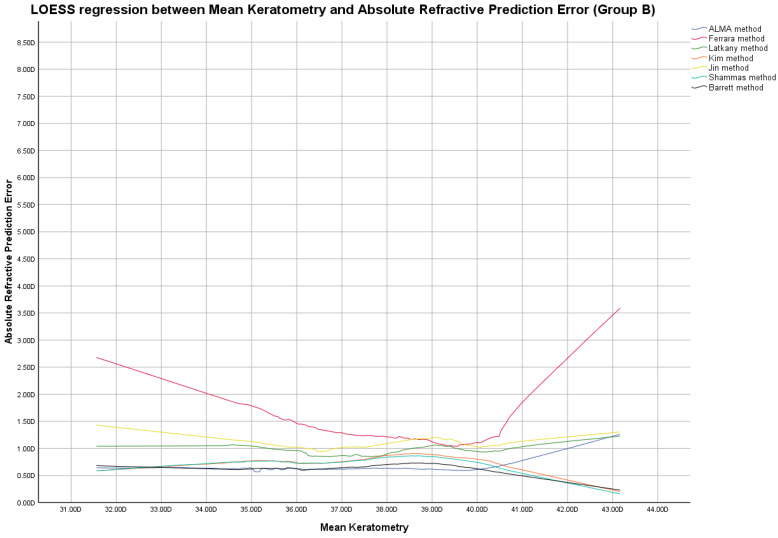
Locally estimated scatterplot smoothing (LOESS) curve between refractive prediction errors generated for each examined formula and the related mean keratometry value. (Group B).

**Figure 5:**
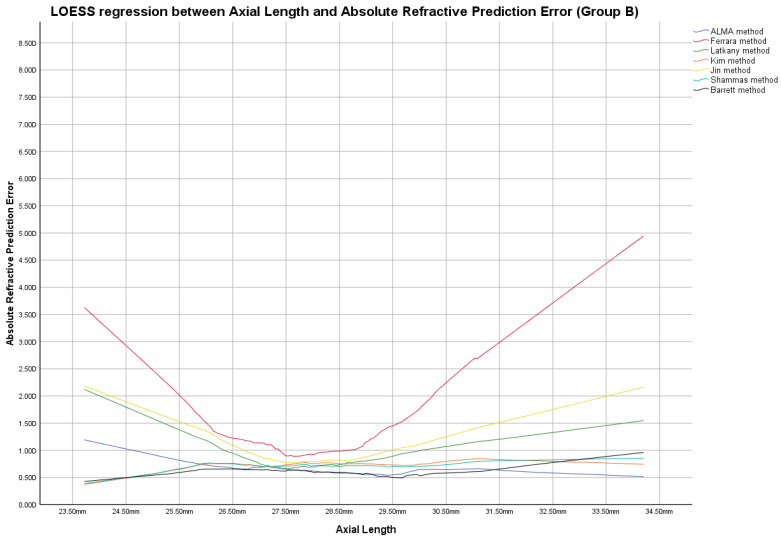
Locally estimated scatterplot smoothing (LOESS) curve between refractive prediction errors generated for each examined formula and the related axial length value. (Group B).

**Figure 6:**
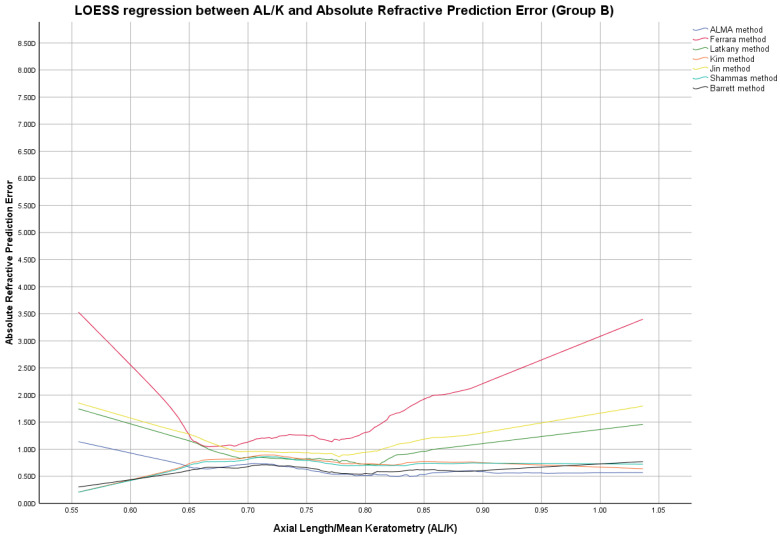
Locally estimated scatterplot smoothing (LOESS) curve between refractive prediction errors generated for each examined formula and the related axial length (AL)/mean keratometry (K) value. (Group B).

In this group, the ME was zeroed out. Modified lens constants obtained by zeroing-out the ME are shown in Supplementary Table S1. Figure 4, Figure 5 and Figure 6 report LOESS regression showing the trend line of PEs according to K values, AL measurements, and AL/K ratio. The relationship among all IOL power calculation methods analyzed for all patients of Group B plotted with the K values, AL measurements, and AL/K ratio are shown in Table 2, Table 3 and Table 4 and Supplementary Table S2. Bonferroni post-hoc analysis and multiple comparisons according to the Cochran Q test for each analyzed range are shown in Supplementary Tables S3 and S4. Boxplot diagrams and bar graphs reporting refractive outcomes obtained in each individual analyzed range were reported in Supplementary Figures S4–S6.

## 4. Discussion

Common guidelines to evaluate IOL power calculation accuracy are crucial: for this reason, since 2015, different protocols have been published [20,21,22]. Hoffer et al. initially pointed out the need to eliminate the systemic bias given by the lens-constant with zeroing-out the ME by adjusting the PE for each method [20]. Hoffer et al. reproposed constant optimization through zeroing-out the mean error in 2020, to eliminate any systematic error arising from the clinical environment, including the biometer, the surgical technique, and the IOL physical properties [21]. Additionally, Holladay et al. gave similar recommendations [22].

Some authors considered it inappropriate to zero-out the ME for atypical eyes [23,44], but Hoffer et al. reported that constant optimization would be preferable when analyzing post-LRS eyes [21].

In addition, other studies that perform zeroing-out of the ME in atypical eyes, e.g., vitrectomized eyes [45] and post-LRS eyes [16,24] have been published.

Due to these discordant opinions, and following the example of other papers [16,24], this study was performed both with and without zeroing-out the ME, strengthening the obtained results. Group A and Group B are partially overlapped but this is not a limitation, because the aim of the study was the analysis of formula accuracy after myopic-LRS with and without constant optimization. Therefore, the groups were almost the same, but they were analyzed in completely different ways. For this purpose, similar database results are required; results should be read in light of the above-mentioned debate [16,21,23,24,44,45].

It is well known that in eyes that did not undergo LRS, it is necessary to utilize different formulas according to different ranges of AL to obtain the best result [46]. This proposal cannot be utilized for post myopic-LRS eyes because they require specific IOL power calculation formulas. The novelty of our study is that not the AL ranges alone, but the combination of AL and K ranges have been considered.

Starting from these concepts, this study aims to verify if different methods of IOL power calculation after myopic-LRS could provide better results in different ranges of the following parameters:

- K, as the K is the value that is modified by the surgery.
- AL.
- AL/K ratio.

The best refractive outcomes obtained by comparing the examined IOL power calculation methods, plotted with the corresponding K and AL values, were reported in the results section and in Figure 1, Figure 2, Figure 4 and Figure 5, Table 2, and Supplementary Tables S2 and S4.

AL/K was studied as an additional parameter to choose the best IOL power calculation formula, as shown in Figure 3 and Figure 6, Table 3, and Supplementary Tables S2 and S3.

On this basis, a multi-formula approach, based on different K readings and AL measurements and summarized in Table 5, both utilizing manufacturers’ suggested A-constant (Group A) and modified A-constant through zeroing-out the ME (Group B) has been proposed. In case more than one formula is suggested, AL/K could clarify which should be preferred. If the AL/K is also inconclusive, the average between the two IOL power values could be utilized. In Group B, when K ≤ 38.00 D and AL > 29.50 mm ALMA and Barrett-TK methods are equivalent but, considering that in these ranges the AL/K parameter is always greater than 0.75, the ALMA formula is preferred.

The adoption of the PE as the primary outcome instead of IOL prediction error is a point of strength of this study. In fact, according to Hoffer et al., converting IOL prediction errors to PEs using a constant factor over the entire AL range is an error because this factor changes as a function of ocular parameters [21,47]. Whereas most of the recent studies regarding IOL power calculation after LRS are based on the ASCRS IOL calculator [18,19] where only the differences between ASCRS-suggested IOL powers and the implanted IOL powers could be calculated.

Another point of strength of this study is the analysis of the Barrett-TK through zeroing-out the ME, which was obtained with a specific computer programming language, following the suggestion proposed by Hoffer et al. [21].

One limitation of this study is that out of 132 eyes, zeroing-out the ME was only possible in 90 eyes due to the presence of a small number of patients for some IOL models or because the IOL model was unknown. The low number of patients for some IOL models could cause an alteration of the A-constant, due to an outlier. Consequently, zeroing-out the ME could mute these outliers. Nonetheless, the use of MedAE instead of the absolute ME and the presence of multiple IOL models could limit the influence of eventual outliers.

Other limitations are the study retrospective design and the presence of some patients’ data derived from literature [26,26], but that information was not included in Group B.

To obtain a large database in studies regarding IOL power calculation after myopic-LRS is difficult. When limited data are available, analyzing more than one IOL model is appropriate [21]. In addition, multiple IOL models were analyzed in other recent studies regarding IOL power calculation accuracy after myopic-LRS [16,17,18,24,30]. In fact, multicenter nature of the study was justified by the extreme difficulty to obtain a large and reliable database of post-refractive surgery eyes that underwent cataract surgery.

This study was performed with zeroing-out the ME for each IOL model to minimize the bias given by the implementation of multiple IOL models, as per Hoffer protocols [20]. In addition, constants optimization achieved through zeroing-out the MEs eliminates any systematic error, making all formulas comparable.

A multi-formula approach according to different ranges of AL and K could be applied also to “normal” eyes or “special” eyes (e.g., keratoconus eyes), but they require different IOL power calculation formulas.

In conclusion, considering the K, AL, and AL/K parameters, it is possible to utilize a multi-formula approach by choosing a specific method that could give better outcomes in certain subpopulations, as shown in Table 5. Even if larger studies utilizing different IOL models are needed, the multi-formula approach could represent the future answer to improve refractive results in post myopic-LRS eyes.

## Figures and Tables

**Table 1:** Comparison of refractive outcome among examined formulas (Group A and Group B).

	ALMA	Barrett	Ferrara	Jin	Kim	Latkany	Shammas
Parameter	Group A (132 eyes)						
MedAE	0.81 D	0.79 D	2.01 D	1.19 D	1.22 D	2.32 D	0.88 D
MAE/STD	0.99/0.07 D	0.96/0.07 D	2.39/0.15 D	1.51/0.09 D	1.46/0.09 D	2.41/0.12 D	1.02/0.07 D
Min-Max	0.01/4.43 D	0.01/4.89 D	0.00/8.22 D	0.01/4.75 D	0.01/6.36 D	0.02/6.62 D	0.00/4.87 D
IQR	1.06 D	1.08 D	2.32 D	1.48 D	1.49 D	1.91 D	1.08 D
CI 95%	0.85–1.13 D	0.82–1.10 D	2.11–2.69 D	1.33–1.69 D	1.28–1.64 D	2.17–2.65 D	0.88–1.16 D
<0.5 D (%)	53 (40.2%)	51 (38.6%)	11 (8.3%)	30 (22.7%)	30 (22.7%)	14 (10.6%)	51 (38.6%)
<1.0 D (%)	87 (65.9%)	88 (66.7%)	38 (28.8%)	60 (45.5%)	60 (45.5%)	27 (20.5%)	82 (62.1%)
	Group B (90 eyes)						
MedAE	0.60 D	0.52 D	1.27 D	1.08 D	0.63 D	0.86 D	0.61 D
MAE/STD	0.74/0.06 D	0.67/0.06 D	1.66/0.14 D	1.14/0.08 D	0.80/0.07 D	1.04/0.08 D	0.78/0.06 D
Min-Max	0.00–2.47 D	0.02/2.34 D	0.03/5.08 D	0.02/2.81 D	0.00/2.83 D	0.01/2.91 D	0.03/2.65 D
IQR/CI95%	0.89 D	0.85 D	1.85 D	1.23 D	0.97 D	1.12 D	0.94 D
CI 95%	0.61–0.86 D	0.56–0.78 D	1.39–1.93 D	0.98–1.30 D	0.67–0.93 D	0.70–1.02 D	0.65–0.91 D
<0.5 D (%)	46 (51.1%)	51 (56.7%)	23 (25.6%)	29 (32.3%)	45 (50.0%)	35 (37.8%)	46 (51.1%)
<1.0 D (%)	68 (75.6%)	69 (76.7%)	43 (47.8%)	47 (52.2%)	62 (68.9%)	53 (58.9%)	66 (73.3%)

MedAE: median absolute error; MAE: mean absolute error; STD: standard error; Min-Max: minimum and maximum errors; IQR: interquartile range; CI 95%: 95% confidence interval around the mean; <0.5 D (%)/<1.0 D (%): numbers and percentages of eyes with a refractive prediction error (PE) within 0.50 D and within 1.00 D.

**Table 2:** Comparison of refractive outcome among examined formulas according to different mean keratometry (K) ranges. (Group A and B).

G	Formula	ALMA	Barrett	Ferrara	Jin	Kim	Latkany	Shammas
	K ≤ 36.0 D (Group A = 28 eyes/Group B = 24 eyes)							
A	MedAE	1.08 D	0.80 D	3.74 D	2.02 D	0.92 D	3.09 D	1.10 D
	<0.5 D (%)	7 (25.0%)	11 (39.3%)	1 (3.6%)	2 (7.1%)	11 (39.3%)	1 (3.6%)	8 (28.6%)
	<1.0 D (%)	14 (50.0%)	18 (64.3%)	2 (7.1%)	9 (32.1%)	17 (60.7%)	1 (3.6%)	15 (53.6%)
B	MedAE	0.54 D	0.41 D	1.89 D	1.27 D	0.57 D	1.01 D	0.63 D
	<0.5 D (%)	15 (62.5%)	14 (58.3%)	3 (12.5%)	8 (33.3%)	13 (54.2%)	9 (37.5%)	14 (58.3%)
	<1.0 D (%)	18 (75.0%)	19 (79.2%)	8 (33.3%)	11 (45.8%)	17 (70.8%)	12 (50.0%)	19 (79.2%)
	36.0 D < K ≤ 38.0 D (Group A = 32 eyes/Group B =23 eyes)							
A	MedAE	0.94 D	0.74 D	2.12 D	1.47 D	1.34 D	2.64 D	0.71 D
	<0.5 D (%)	12 (37.5%)	11 (34.4%)	2 (6.3%)	8 (25.0%)	7 (21.9%)	2 (6.3%)	14 (43.8%)
	<1.0 D (%)	20 (62.5%)	24 (75.0%)	7 (21.9%)	13 (40.6%)	12 (37.5%)	6 (18.8%)	22 (68.8%)
B	MedAE	0.70 D	0.60 D	1.28 D	0.99 D	0.61 D	0.83 D	0.56 D
	<0.5 D (%)	11 (47.8%)	12 (52.2%)	6 (26.1%)	7 (30.4%)	12 (52.2%)	8 (34.8%)	12 (52.2%)
	<1.0 D (%)	19 (82.6%)	17 (73.9%)	11 (47.8%)	16 (69.6%)	16 (69.6%)	17 (73.9%)	16 (69.6%)
	38.0 D < K ≤ 40.0 D (Group A = 41 eyes/Group B = 26 eyes)							
A	MedAE	0.83 D	0.92 D	1.73 D	1.14 D	1.28 D	2.17 D	1.02 D
	<0.5 D (%)	15 (36.6%)	13 (31.7%)	4 (9.8%)	12 (29.3%)	10 (24.4%)	4 (9.8%)	14 (34.1%)
	<1.0 D (%)	27 (65.9%)	24 (58.5%)	12 (29.3%)	19 (46.3%)	17 (41.5%)	8 (19.5%)	24 (58.5%)
B	MedAE	0.44 D	0.66 D	0.99 D	1.18 D	0.79 D	1.02 D	0.74 D
	<0.5 D (%)	14 (53.8%)	13 (50.0%)	9 (34.6%)	7 (26.9%)	10 (38.5%)	9 (34.6%)	10 (38.5%)
	<1.0 D (%)	20 (76.9%)	17 (65.4%)	17 (65.4%)	12 (46.2%)	15 (57.7%)	14 (53.8%)	16 (61.5%)
	K > 40.0 D (Group A = 31 eyes/Group B = 17 eyes)							
A	MedAE	0.53 D	0.60 D	1.06 D	0.95 D	1.33 D	1.44 D	0.76 D
	<0.5 D (%)	19 (61.3%)	16 (51.6%)	4 (12.9%)	8 (25.8%)	5 (16.1%)	7 (22.6%)	15 (48.4%)
	<1.0 D (%)	26 (83.9%)	22 (71.0%)	17 (54.8%)	19 (61.3%)	14 (45.2%)	12 (38.7%)	21 (67.7%)
B	MedAE	0.81 D	0.22 D	2.16 D	1.10 D	0.33 D	1.06 D	0.27 D
	<0.5 D (%)	6 (35.3%)	12 (70.6%)	5 (29.4%)	7 (41.2%)	10 (58.8%)	8 (47.1%)	12 (70.6%)
	<1.0 D (%)	11 (64.7%)	16 (94.1%)	7 (41.2%)	9 (52.9%)	14 (82.4%)	10 (58.8%)	15 (88.2%)

G: Group; MedAE: median absolute error; <0.5 D (%)/<1.0 D (%): numbers and percentages of eyes with a refractive prediction error (PE) within 0.50 D and within 1.00 D.

**Table 3:** Comparison of refractive outcome among examined formulas according to different axial length (AL), ranges. (Groups A and B).

G	Formula	ALMA	Barrett	Ferrara	Jin	Kim	Latkany	Shammas
	AL ≤ 26.5 mm (Group A = 47 eyes/Group B = 24 eyes)							
A	MedAE	0.56 D	0.64 D	0.98 D	0.85 D	0.98 D	1.36 D	0.71 D
	<0.5 D (%)	26 (55.3%)	23 (48.9%)	9 (19.1%)	16 (34.0%)	14 (29.8%)	11 (23.4%)	23 (48.9%)
	<1.0 D (%)	33 (70.2%)	34 (72.3%)	27 (57.4%)	32 (68.1%)	28 (59.6%)	21 (44.7%)	32 (68.1%)
B	MedAE	0.80 D	0.32 D	1.99 D	1.49 D	0.29 D	1.25 D	0.34 D
	<0.5 D (%)	9 (37.5%)	15 (62.5%)	7 (29.2%)	2 (8.3%)	14 (58.3%)	5 (20.8%)	14 (58.3%)
	<1.0 D (%)	16 (66.7%)	17 (70.8%)	8 (33.3%)	9 (37.5%)	16 (66.7%)	10 (41.7%)	16 (66.7%)
	26.50 < AL ≤ 28.00 mm (Group A = 32 eyes/Group B = 22 eyes)							
A	MedAE	0.83 D	0.92 D	1.86 D	1.01 D	1.40 D	2.35 D	1.02 D
	<0.5 D (%)	11 (34.4%)	11 (34.4%)	1 (3.1%)	10 (31.1%)	8 (25.0%)	3 (9.4%)	10 (31.1%)
	<1.0 D (%)	23 (71.9%)	20 (62.5%)	9 (28.1%)	18 (56.3%)	13 (40.6%)	6 (18.8%)	18 (56.3%)
B	MedAE	0.51 D	0.64 D	0.95 D	0.68 D	0.68 D	0.48 D	0.68 D
	<0.5 D (%)	12 (54.5%)	11 (50.0%)	7 (31.8%)	11 (50.0%)	10(45.5%)	13 (59.1%)	10 (45.5%)
	<1.0 D (%)	17 (77.3%)	18 (81.8%)	15 (68.2%)	15 (68.2%)	15 (68.2%)	17 (77.3%)	17 (77.3%)
	28.00 < AL ≤ 29.50 mm (Group A = 29 eyes/Group B = 23 eyes)							
A	MedAE	0.99 D	0.98 D	2.76 D	1.89 D	1.75 D	2.99 D	0.94 D
	<0.5 D (%)	9 (31.0%)	9 (31.0%)	1 (3.4%)	3 (10.3%)	5 (17.2%)	0 (0.0%)	10 (34.5%)
	<1.0 D (%)	18 (62.1%)	17 (58.6%)	2 (6.9%)	5 (17.2%)	7 (24.1%)	0 (0.0%)	19 (65.5%)
B	MedAE	0.57 D	0.53 D	0.99 D	0.82 D	0.68 D	0.69 D	0.58 D
	<0.5 D (%)	13 (56.6%)	14 (60.9%)	5 (21.7%)	11 (47.8%)	11 (47.8%)	10 (43.5%)	13 (56.6%)
	<1.0 D (%)	19 (82.6%)	17 (73.9%)	15 (65.2%)	15 (65.2%)	16 (69.6%)	16 (69.6%)	17 (73.9%)
	AL > 29.50 mm (Group A = 24 eyes/Group B = 21 eyes)							
A	MedAE	1.05 D	0.82 D	4.96 D	2.26 D	1.10 D	3.20 D	0.85 D
	<0.5 D (%)	7 (29.2%)	8 (33.3%)	0 (0.0%)	1(4.2%)	6 (25.0%)	0 (0.0%)	8 (33.3%)
	<1.0 D (%)	13 (54.2%)	17 (70.8%)	0 (0.0%)	5 (20.8%)	12 (50.0%)	0 (0.0%)	13 (54.2%)
B	MedAE	0.56 D	0.41 D	2.43 D	1.53 D	0.68 D	1.28 D	0.67 D
	<0.5 D (%)	12 (57.1%)	11 (52.4%)	4 (19.0%)	5 (23.8%)	10 (47.6%)	6 (28.6%)	9 (42.9%)
	<1.0 D (%)	16 (76.2%)	17 (81.0%)	5 (23.8%)	8 (38.1%)	15 (71.4%)	10 (47.6%)	16 (76.2%)

G: group; MedAE: median absolute error; <0.5 D (%)/<1.0 D (%): numbers and percentages of eyes with a refractive prediction error within 0.50 D and within 1.00 D.

**Table 4:** Comparison of refractive outcome among examined formulas according to different axial length/mean keratometry ratio (AL/K) ranges.

G	Formula	ALMA	Barrett	Ferrara	Jin	Kim	Latkany	Shammas
	AL/K ≤ 0.67 (Group A = 40 eyes/Group B = 20 eyes)							
A	MedAE	0.53 D	0.62 D	1.01 D	0.89 D	1.03 D	1.38 D	0.74 D
	<0.5 D (%)	22 (55.0%)	20 (50.0%)	8 (20.0%)	13 (32.5%)	11 (27.5%)	10 (25.0%)	19 (47.5%)
	<1.0 D (%)	30 (75.0%)	29 (72.5%)	23 (57.5%)	26 (65.0%)	23 (57.5%)	18 (45.0%)	28 (70.5%)
B	MedAE	0.78 D	0.32 D	1.39 D	1.45 D	0.29 D	1.25 D	0.26 D
	<0.5 D (%)	8 (40.0%)	13 (65.0%)	6 (30.0%)	3 (15.0%)	12 (60.0%)	5 (25.0%)	13 (65.0%)
	<1.0 D (%)	13 (65.0%)	16 (80.0%)	9 (45.0%)	7 (35.0%)	16 (80.0%)	8 (40.0%)	16 (80.0%)
	0.67 < AL/K ≤ 0.75 (Group A = 40 eyes/Group B = 26 eyes)							
A	MedAE	0.78 D	0.95 D	1.73 D	1.24 D	1.47 D	2.51 D	1.02 D
	<0.5 D (%)	17 (42.5%)	1 (2.5%)	2 (5.0%)	6 (15.0%)	11 (27.5%)	15 (37.5%)	12 (30.0%)
	<1.0 D (%)	17 (42.5%)	12 (30.0%)	1 (2.5%)	11 (27.5%)	6 (15.0%)	2 (5.0%)	15 (37.5%)
B	MedAE	0.78 D	0.70 D	1.02 D	0.98 D	0.77 D	0.61 D	0.70 D
	<0.5 D (%)	11 (42.3%)	12 (46.2%)	9 (34.6%)	12 (46.2%)	10 (38.5%)	13 (50.0%)	11 (42.3%)
	<1.0 D (%)	18 (69.2%)	18 (69.2%)	15 (57.7%)	16 (61.5%)	14 (53.8%)	17 (65.4%)	16 (61.5%)
	AL/K > 0.75 (Group A = 52 eyes/Group B = 44 eyes)							
A	MedAE	1.02 D	0.76 D	3.53 D	2.04 D	1.17 D	2.99 D	0.87 D
	<0.5 D (%)	14 (26.9%)	19 (36.5%)	2 (3.8%)	6 (11.5%)	16 (30.8%)	2 (3.8%)	17 (32.7%)
	<1.0 D (%)	28 (53.8%)	35 (67.3%)	4 (7.7%)	15 (28.8%)	25 (48.1%)	3 (5.8%)	32 (61.5%)
B	MedAE	0.55 D	0.50 D	1.30 D	0.98 D	0.57 D	0.80 D	0.63 D
	<0.5 D (%)	27 (61.4%)	26 (59.1%)	8 (18.2%)	14 (31.8%)	23 (52.3%)	16 (36.4%)	22 (50.0%)
	<1.0 D (%)	37 (84.1%)	35 (79.5%)	19 (43.2%)	24 (54.5%)	32 (72.7%)	28 (63.6%)	34 (77.3%)

G: group; MedAE: median absolute error; <0.5 D (%)/<1.0 D (%): numbers and percentages of eyes with a refractive prediction error within 0.50 D and within 1.00 D.

**Table 5:** Suggested formulas plotted by different mean keratometry (K) ranges and different axial length (AL) ranges. In case of two formulas have been suggested, AL/K could clarify which should be preferred. If the analysis of the AL/K parameter is inconclusive, it is possible to consider an average IOL power value between the two proposed methods.

	Group A			
Parameters	AL ≤ 26.5 mm	26.5 mm < AL ≤ 28.0 mm	28.0 mm < AL ≤ 29.5 mm	AL > 29.5 mm
K ≤ 36.0 D	Barrett	Barrett	Barrett	Barrett
36.0 D < K ≤ 38.0 D	ALMA	ALMA/Barrett	ALMA/Barrett	Barrett
38.0 D < K ≤ 40.0 D	ALMA	ALMA	ALMA	ALMA/Barrett
K > 40.0 D	ALMA	ALMA	ALMA	ALMA/Barrett
	Group B			
Parameters	AL ≤ 26.5 mm	26.5 mm < AL ≤ 28.0 mm	28.0 mm < AL ≤ 29.5 mm	AL > 29.5 mm
K ≤ 36.0 D	Barrett	ALMA/Barrett	ALMA	ALMA
36.0 D < K ≤ 38.0 D	Barrett	ALMA/Barrett	ALMA	ALMA
38.0 D < K ≤ 40.0 D	ALMA/Barrett	ALMA	ALMA	ALMA/Barrett
K > 40.0 D	Barrett	Barrett	ALMA/Barrett	Barrett
	Group A		Group B	
AL/K ≤ 0.67	ALMA		Barrett	
0.67 < AL/K ≤ 0.75	ALMA		-	
AL/K > 0.75	Barrett		ALMA	

K: mean keratometry; AL: axial length.

## Data Availability

The datasets generated and analyzed during the current study are available from the corresponding author upon reasonable request.

## Supplementary Materials

The following supporting information can be downloaded at: https://www.mdpi.com/article/10.3390/jcm12082890/s1, Table S1: Comparison of Refractive Outcome among examined formulas: additional parameters (Group A and B); Table S2: Comparison of Refractive Outcome among examined formulas: additional parameters (Group A and B); Table S3: Multiple comparisons among examined formulas according to Axial Length / Mean Keratometry ratio (AL/K) ranges. (Group A and Group B). Only statistically significant differences; Table S4: Multiple comparisons among examined formulas according to different Axial Length (AL) and Mean Keratometry (K) Ranges. (Group A and Group B). Only statistically significant differences were reported; Figure S1: Box-plot diagrams and bar graphs reporting refractive outcomes for each range of mean keratometry (K) values analysed (Group A); Figure S2: Box-plot diagrams and bar graphs reporting refractive outcomes for each range of axial length (AL) measurements analysed (Group A); Figure S3: Box-plot diagrams and bar graphs reporting refractive outcomes for each range of Axial Length/Mean Keratometry ratio (AL/K) values analysed (Group A); Figure S4: Box-plot diagrams and bar graphs reporting refractive outcomes for each range of mean keratometry (K) values analysed (Group B); Figure S5: Box-plot diagrams and bar graphs reporting refractive outcomes for each range of axial length (AL) measurements analysed (Group B); Figure S6: Box-plot diagrams and bar graphs reporting refractive outcomes for each range of Axial Length/Mean Keratometry ratio (AL/K) values analysed (Group B).
