# Supplementary material for: A No-History Multi-Formula Approach to Improve the IOL Power Calculation after Laser Refractive Surgery: Preliminary Results
Source: J Clin Med. 2023 Apr 15;12(8):2890. doi: 10.3390/jcm12082890 (PMC10144756; doi:10.3390/jcm12082890)
Supplement: Supplementary file 1 [file jcm-12-02890-s001.zip › Supplementary Tables.pdf]

Supplementary Tables

Supplementary Table S1. P-values, number of different IOL models involved in the study and A-constant before and after zeroing out the mean error for all examined formulas. (Group A and Group B). Only statistically significant differences were reported.

Formula	Group A	Group B
ALMA	F: p1=<0.001/p2<0.001/p3<0.001<br>J: p1<0.001/p2=0.001/p3<0.001<br>K: p1<0.001/p2=0.004/p3<0.001<br>L: p1<0.001/p2<0.001/p3<0.001	F: p1<0.001/p2<0.001/p3<0.001<br>J: p1<0.001/p2=0.005/p3<0.001<br>L: p1=0.002/p2=0.049/p3=0.006
Barrett	F: p1<0.001/p2<0.001/p3<0.001<br>J: p1<0.001/p2=0.002/p3<0.001<br>K: p1<0.001/p2=0.009/p3<0.001<br>L: p1<0.001/p2<0.001/p3<0.001	F: p1<0.001/p2<0.001/p3<0.001<br>J: p1<0.001/p2<0.001/p3<0.001<br>L: p1<0.001/p2=0.005/p3=0.003
Shammas	F: p1<0.001/p2<0.001/p3<0.001<br>J: p1=-/p2=0.002/p3=0.004<br>K: p1=0.001/p2=0.009/p3=0.004<br>L: p1<0.001/p2<0.002/p3<0.001	F: p1<0.001/p2<0.001/p3<0.001<br>J: p1=0.012/p2=0.005/p3<0.001<br>L: p1=-/p2=0.049/p3=0.016
Kim	-	F: p1=0.004/p2<0.001/p3<0.001<br>J: p1=-/p2=0.009/p3=-

IOL Model	N	A-Const.	ALMA	Barrett	Ferrara	Jin	Kim	Latkany	Shammas
**Abbot AAB00 Sensar**	2	118.4	X	X	X	X	X	X	X
**Alcon Acrysof MA60BM**	5	118.9	118.3	120.0	116.2	120.5	120.7	122.1	119.1
**Alcon Restor SA60D3**	2	118.1	X	X	X	X	X	X	X
**Alcon SA60AT**	3	118.4	117.2	119.1	114.9	120.0	120.3	121.8	118.7
**Alcon SN60WF**	4	118.7	118.7	120.4	116.4	120.7	121.3	122.2	119.9
**AMO Sensar AR40e**	14	118.4	116.8	118.0	114.0	119.5	119.3	121.3	117.8
**AMO Tecnis PCB00**	15	118.8	116.5	118.6	112.5	120.6	120.1	122.3	118.7
**AMO Tecnis ZMA00**	2	119.1	X	X	X	X	X	X	X
**AMO Tecnis Z9000**	16	119.0	118.7	120.0	116.3	121.0	120.9	122.9	119.6
**B&L Akreos Adapt**	11	118.0	118.8	120.3	116.8	120.6	121.2	122.6	119.7
**B&L Akreos AOMI60**	2	118.4	X	X	X	X	X	X	X
**B&L C31UB**	1	119.0	X	X	X	X	X	X	X
**Corneal ACR600SE**	2	120.0	X	X	X	X	X	X	X
**Corneal PHACNS5**	1	118.5	X	X	X	X	X	X	X
**Corneal Quatrix**	1	119.6	X	X	X	X	X	X	X
**Curamed SA60CZ**	3	118.8	116.4	119.9	107.1	122.1	121.2	124.4	119.5
**Hexavision HQ203HEP**	2	118.2	X	X	X	X	X	X	X
**Hoya AF1FY60AD**	3	118.4	118.0	119.7	115.3	120.7	121.1	122.0	119.7
**Hoya iSert 250**	5	118.4	116.9	118.5	114.3	120.3	119.9	121.8	118.2
**Hoya VA6BB**	6	118.7	116.3	117.8	112.4	121.1	119.4	122.7	118.1
**Soleko Fil611**	1	119.0	X	X	X	X	X	X	X
**Tech Med ISP60Z**	5	118.2	116.5	117.5	112.8	118.5	119.5	119.7	117.9
**Zeiss CT Spheris 203**	1	118.0	X	X	X	X	X	X	X

Formula: best method compared to the formulas shown in the adjacent columns; F: Ferrara Formula, K: Kim Formula, J; Jin Formula; L: Latkany Formula; p1: level of significance regarding median absolute errors between formulas according to the Friedman test with Bonferroni correction; p2/p3: level of significance regarding the percentages of eyes with a refractive Prediction Error within 0.50D and within 1.00D between formulas, according to Cochran Q Test; - = p value > 0.05; N: Number of IOL analyzed for each model; X: optimized constant not available.

Supplementary Table S2: Comparison of Refractive Outcome among examined formulas: additional parameters (Group A and B)

G	Formula	ALMA	Barrett	Ferrara	Jin	Kim	Latkany	Shammas
	K≤ 36.0D							
**A**	**MAE/STD**	1.37D/0.19D	0.91D/0.14D	3.85D/0.37D	2.12D/0.22D	1.10D/0.16D	3.22D/0.24D	1.03D/0.12D
	**Min/Max**	0.07/3.52D	0.03/2.98D	0.01/8.22D	0.22/3.76D	0.04/2.53D	0.49/5.37D	0.05/3.15D
	**IQR/CI 95%**	1.38/1.00-1.74D	1.12/0.64-1.18D	2.92/3.13-4.59D	2.27/1.68-2.56D	1.64/0.83-1.47D	2.16/2.74-3.68D	0.95/0.79-1.27D
**B**	**MAE/STD**	0.78D/0.14D	0.67D/0.11D	2.05D/0.25D	1.24D/0.18D	0.80D/0.13D	1.12D/0.17D	0.81D/0.12D
	**Min/Max**	0.00/2.47D	0.07/2.16D	0.30/4.38D	0.11/2.72D	0.10/2.66D	0.03/2.64D	0.03/2.65D
	**IQR/CI 95%**	0.79/0.50-1.06D	0.88/0.45-0.89D	2.11/1.55-2.55D	1.57/0.90-1.58D	0.87/0.56-1.04D	1.48/0.80-1.46D	0.72/0.57-1.05D
	36.0D <K ≤ 38.0D							
**A**	**MAE/STD**	0.96D/0.11D	0.86D/0.11D	2.47D/0.29D	1.55D/0.18D	1.34D/0.16D	2.54D/0.23D	0.91D/0.13D
	**Min/Max**	0.02/2.47D	0.01/3.12D	0.00/7.22D	0.02/4.55D	0.01/4.37D	0.02/6.06D	0.09/3.24D
	**IQR/CI 95%**	0.97/0.74 -1.18D	0.73/0.65-1.08D	2.41/1.90-3.04D	1.49/1.19-1.91D	1.06/1.03-1.65D	1.57/2.10-2.98D	0.97/0.66-1.16D
**B**	**MAE/STD**	0.74D/0.11D	0.66D/0.09D	1.56D/0.28D	1.00D/0.14D	0.78D/0.12D	0.93D/0.14D	0.78D/0.11D
	**Min/Max**	0.08/2.16D	0.08/1.43D	0.03/5.08D	0.03/2.42D	0.00/1.76D	0.01/2.51D	0.14/1.75D
	**IQR/CI 95%**	0.71/0.52-0.96D	0.85/0.48-0.84D	1.73/1.02-2.10D	1.01/0.73-1.27D	1.11/0.54-1.02D	0.84/0.65-1.21D	0.88/0.56-1.00D
	38.0D < K ≤ 40.0D							
**A**	**MAE/STD**	0.92D/0.11D	1.06D/0.12D	1.95D/0.20D	1.370/0.15D	1.54D/0.17D	2.22D/0.19D	1.09D/0.12D
	**Min/Max**	0.01/2.79D	0.05/2.74D	0.27/6.08D	0.01/3.10D	0.03/3.50D	0.11/4.43D	0.04/2.43D
	**IQR/CI 95%**	1.08/0.71-1.13D	1.19/0.83-1.29D	1.80/1.56-2.34D	1.42/1.01-1.59D	1.80/1.21-1.87D	1.90/1.85-2.59D	1.38/0.86-1.32D
**B**	**MAE/STD**	0.66D/0.12D	0.83D/0.12D	1.19D/0.19D	1.27D/0.15D	1.00D/0.14D	1.14D/0.15D	0.95D/0.14D
	**Min/Max**	0.01/2.07D	0.02/2.34D	0.18/4.03D	0.16/2.81D	0.10/2.83D	0.17/2.91D	0.03/2.62D
	**IQR/CI 95%**	0.95/0.43-0.89D	1.04/0.59-1.07D	1.21/0.81-1.57D	1.35/0.98-1.56D	1.01/0.72-1.28D	1.20/0.85-1.43D	1.11/0.68-1.22D
	K > 40.0D							
**A**	**MAE/STD**	0.79D/0.17D	0.95D/0.20D	1.60D/0.21D	1.19D/0.18D	1.75D/0.24D	1.80D/0.27D	1.03D/0.20D
	**Min/Max**	0.02/4.43D	0.00/4.89D	0.28/5.42D	0.05/4.75D	0.25/6.36D	0.02/6.62D	0.01/4.87D
	**IQR/CI 95%**	0.81/0.46-1.12D	1.26/0.56-1.34D	1.52/1.20-2.00D	0.82/0.83-1.55D	1.44/1.28-2.22D	1.74/1.27-2.33D	1.22/0.63-1.43D
**B**	**MAE/STD**	0.77D/0.12D	0.43D/0.09D	1.98D/0.39D	1.02D/0.20D	0.53D/0.12D	0.95D/0.19D	0.46D/0.11D
	**Min/Max**	0.01/1.37D	0.02/1.47D	0.07/4.28D	0.02/2.33D	0.02/1.56D	0.01/2.42D	0.04/1.56D
	**IQR/CI 95%**	0.89/0.54-1.00D	0.49/0.25-0.62D	3.38/1.23-2.73D	1.61/0.64-1.41D	0.66/0.29-0.77D	1.32/0.58-1.32D	0.62/0.24-0.68D
	AL ≤ 26.5mm							
**A**	**MAE/STD**	0.83D/0.12D	0.86D/0.13D	1.21D/0.11D	1.01D/0.13D	1.20D/0.16D	1.59D/0.19D	0.98D/0.14D
	**Min/Max**	0.02/4.43D	0.01/4.89D	0.00/3.22D	0.01/4.75D	0.01/6.36D	0.02/6.62D	0.03/4.87D
	**IQR/CI 95%**	0.91/0.60-1.06D	0.87/0.61-1.11D	0.96/1.00-1.42D	0.70/0.76-1.26D	1.00/0.89-1.51D	1.45/1.22-1.96D	1.14/0.70-1.26D
**B**	**MAE/STD**	0.82D/0.10D	0.61D/0.12D	1.98D/0.30D	1.53D/0.15D	0.74D/0.16D	1.36D/0.16D	0.76D/0.16D
	**Min/Max**	0.02/1.85D	0.02/1.77D	0.07/4.28D	0.30/2.81D	0.02/2.66D	0.15/2.91D	0.03/2.65D
	**IQR/CI 95%**	0.88/0.62-1.02D	1.03/0.38-0.83D	2.90/1.39-2.57D	1.08/1.24-1.82D	1.27/0.43-1.05D	1.26/1.06-1.67D	1.13/0.45-1.07D
	26.50 <AL ≤ 28.00mm							
**A**	**MAE/STD**	1.04D/0.15D	1.03D/0.15D	1.90D/0.16D	1.20D/0.16D	1.58D/0.19D	2.38D/0.21D	1.05D/0.13D
	**Min/Max**	0.01/3.17D	0.00/3.40D	0.028/3.64D	0.02/3.64D	0.07/4.81D	0.02/4.27D	0.01/3.54D
	**IQR/CI 95%**	1.02/0.74-1.34D	1.24/0.73-1.32D	1.59/1.59-2.22D	1.39/0.88-1.52D	1.76/1.20-1.57D	1.65/1.20-1.96D	1.12/0.79-1.31D
**B**	**MAE/STD**	0.76D/0.15D	0.68D/0.09D	1.15D/0.21D	0.75D/0.13D	0.76D/0.10D	0.72D/0.14D	0.75D/0.10D
	**Min/Max**	0.01/2.47D	0.14/1.39D	0.18/3.68D	0.02/2.14D	0.11/1.61D	0.01/2.47D	0.03/1.71D
	**IQR/CI 95%**	1.00/0.47-1.05D	0.65/0.51-0.85D	0.90/0.74-1.56D	1.04/0.50-1.00D	0.84/0.56-0.96D	0.95/0.45-0.99D	0.77/0.55-0.95D
	28.00 <AL ≤ 29.50mm							
**A**	**MAE/STD**	0.98D/0.12D	1.11D/0.15D	2.77D/0.23D	1.94D/0.19D	1.85D/0.21D	3.08D/0.22D	1.10D/0.15D
	**Min/Max**	0.02/2.39D	0.06/3.12D	0.40/5.42D	0.01/4.55D	0.04/4.37D	1.31/6.06D	0.05/3.24D
	**IQR/CI 95%**	0.84/0.74-1.22D	1.20/0.81-1.41D	1.70/2.32-3.22D	1.54/1.57-2.31D	1.46/1.44-2.26D	1.52/2.65-3.51D	1.08/0.80-1.40D
**B**	**MAE/STD**	0.65D/0.11D	0.72D/0.13D	1.11D/0.15D	0.87D/0.14D	0.87D/0.15D	0.86D/0.15D	0.79D/0.14D
	**Min/Max**	0.01/2.07D	0.02/2.34D	0.08/2.81D	0.03/2.06D	0.10/2.83D	0.01/2.63D	0.17/2.62D
	**IQR/CI 95%**	0.88/0.43-0.87D	0.84/0.47-0.97D	0.63/0.82-1.42D	0.91/0.60-1.36D	0.85/0.58-1.16D	0.94/0.57-1.15D	0.94/0.52-1.06D
	AL > 29.50mm							
**A**	**MAE/STD**	1.29D/0.20D	0.85D/0.12D	4.94D/0.31D	2.35D/0.22D	1.33D/0.17D	3.25D/0.24D	0.93D/0.12D
	**Min/Max**	0.07/3.52D	0.01/2.28D	2.01/8.22D	0.22/3.76D	0.04/2.94D	1.24/5.19D	0.04/2.06D
	**IQR/CI 95%**	1.41/0.89-1.69D	0.99/0.61-1.09D	2.36/4.33-5.55D	1.95/1.93-2.77D	1.55/0.99-1.67D	2.16/2.78-3.72D	1.05/0.70-1.16D
**B**	**MAE/STD**	0.72D/0.14D	0.67D/0.12D	2.43D/0.32D	1.41D/0.18D	0.84D/0.11D	1.22D/0.17D	0.81D/0.11D
	**Min/Max**	0.00/2.06D	0.07/2.16D	0.03/5.08D	0.09/2.72D	0.00/1.80D	0.07/2.64D	0.08/1.75D
	**IQR/CI 95%**	0.75/0.45-0.99D	0.93/0.43-0.91D	0.99/1.81-3.05D	1.33/1.06-1.76D	0.88/0.62-1.06D	1.21/0.88-1.56D	0.68/0.60-1.02D
	AL/K ≤ 0.67							
**A**	**MAE/STD**	0.84D/0.14D	0.90D/0.16D	1.31D/0.13D	1.07D/0.15D	1.40D/0.20D	1.58D/0.20D	0.99D/0.16D
	**Min/Max**	0.02/4.43D	0.00/4.89D	0.27/3.22D	0.01/4.75D	0.03/6.36D	0.02/6.62D	0.01/4.87D
	**IQR/CI 95%**	0.99/0.56-1.12D	0.96/0.59-1.12D	1.08/1.06-1.56D	1.08/0.77-1.37D	1.35/1.00-1.79D	1.53/1.15-2.01D	1.19/0.67-1.31D
**A**	**MAE/STD**	0.76D/0.11D	0.56D/0.12D	1.87D/0.35D	1.46D/0.18D	0.58D/0.14D	1.33D/0.19D	0.57D/0.14D
	**Min/Max**	0.02/1.56D	0.02/1.77D	0.07/4.28D	0.09/2.81D	0.02/1.90D	0.01/2.91D	0.03/1.98D
	**IQR/CI 95%**	0.94/0.55-0.97D	0.58/0.33-0.79D	3.20/1.19-2.55D	1.29/1.11-1.81D	0.76/0.30-0.86D	1.50/0.96-1.70D	0.79/0.29-0.85D
	0.67 < AL/K ≤ 0.75							
**A**	**MAE/STD**	0.84D/0.10D	1.07D/0.11D	1.89D/0.17D	1.34D/0.12D	1.70D/0.16D	2.46D/0.18D	1.08D/0.12D
	**Min/Max**	0.01/2.79D	0.05/2.70D	0.00/5.42D	0.05/3.10D	0.01/3.82D	0.02/4.37D	0.09/2.76D
	**IQR/CI 95%**	0.93/0.65-1.05D	1.06/0.85-1.29D	1.58/1.56-2.22D	1.30/1.07-1.61D	1.59/1.39-2.01D	1.67/2.12-2.80D	1.39/0.89-1.32D
**B**	**MAE/STD**	0.80D/0.12D	0.79D/0.11D	1.28D/0.20D	0.92D/0.14D	1.00D/0.13D	0.88D/0.14D	0.95D/0.13D
	**Min/Max**	0.01/2.07D	0.02/2.34D	0.18/3.86D	0.02/2.14D	0.14/2.83D	0.03/2.47D	0.19/2.62D
	**IQR/CI 95%**	1.07/0.65-1.04D	0.90/0.57-1.01D	1.57/0.89-1.67D	1.09/0.65-1.19D	1.03/0.73-1.27D	0.94/0.61-1.15D	1.04/0.70-2.00D
	AL/K > 0.75							
**A**	**MAE/STD**	1.24D/0.12D	0.91D/0.10D	3.63D/0.26D	1.97D/0.16D	1.31D/0.13D	3.00D/0.18D	0.99D/0.09D
	**Min/Max**	0.02/3.52D	0.01/3.12D	0.01/8.22D	0.01/4.55D	0.04/4.37D	0.49/6.06D	0.04/3.24D
	**IQR/CI 95%**	1.25/1.00-1.48D	1.05/0.72-1.10D	2.84/3.13-4.13D	1.92/1.66-2.28D	1.49/1.06-1.58D	1.86/2.66-3.36D	0.97/0.81-1.17D
**B**	**MAE/STD**	0.69D/0.09D	0.65D/0.08D	1.79D/0.20D	1.13D/0.12D	0.78D/0.09D	1.00D/0.11D	0.77D/0.08D
	**Min/Max**	0.00/2.7D	0.07/2.16D	0.03/5.08D	0.03/2.72D	0.00/2.667D	0.01/2.64D	0.03/2.65D
	**IQR/CI 95%**	0.58/0.50-0.86D	0.76/0.50-0.80D	1.91/1.41-2.17D	1.09/0.90-1.36D	0.79/0.61-0.95D	1.06/0.78-1.22D	0.74/0.61-0.93D

G: Group; MedAE; MAE: Mean Absolute Error; STD: Standard Error; Min/Max: Minimum and Maximum Errors; IQR: Interquartile Range; CI 95%: 95% Confidence Interval around the mean.

Supplementary Table S3. Multiple comparisons among examined formulas according to Axial Length / Mean Keratometry ratio (AL/K) ranges. (Group A and Group B). Only statistically significant differences were reported.

Formula	AL/K ≤ 0.67	0.67 < AL/K ≤ 0.75	AL/K > 0.75
Group A			
ALMA	F: p1=0.001/p2=0.01/p3=ns<br>J: p1=ns/p2=0.030/p3=ns<br>K: p1=0.013/p2=0.008/p3=ns<br>L: p1=0.003/p2=0.004/p3=0.001	F: p1<0.001/p2<0.001/p3<0.001<br>J: p1=ns/p2=ns/p3=0.021<br>K: p1<0.001/p2=0.002/p3<0.001<br>L: p1<0.001/p2<0.001/p3<0.001	F: p1<0.001/p2=0.004/p3<0.001<br>J: p1=ns/p2=ns/p3=0.010<br>L: p1<0.001/p2=0.004/p3<0.001
Barrett	F: p1=0.003/p2=0.004/p3=ns<br>K: p1=0.028/p2=0.030/p3=ns<br>L: p1=0.006/p2=0.016/p3=0.003	F: p1=0.011/p2=0.002/p3=0.003<br>K: p1=0.007/p2=ns/p3=0.007<br>L: p1<0.001/p2=0.006/p3<0.001	F: p1<0.001/p2<0.001/p3<0.001<br>J: p1<0.001/p2=0.002/p3<0.001<br>K: p1=ns/p2=ns/p3=0.047<br>L: p1<0.001/p2<0.001/p3<0.001
Kim	ns	ns	F: p1<0.001/p2=0.001/p3<0.001<br>J: p1=ns/p2=0.018/p3=0.047<br>L: p1<0.001/p2=0.001/p3<0.001
Jin	L: p1=ns/p2=ns/p3=0.031	F: p1=ns/p2=0.006/p3=ns<br>L: p1<0.001/p2=0.013/p3=0.003	F: p1=0.019/p2=ns/p3=0.029<br>L: p1=0.002/p2=ns/p3=0.017
Shammas	F: p1=ns/p2=0.008/p3=ns<br>L: p1=ns/p2=0.030/p3=0.007	F: p1=0.013/p2<0.001/p3=0.011<br>K: p1=0.010/p2=0.013/p3=0.021<br>L: p1<0.001/p2<0.001/p3<0.001	F: p1<0.001/p2<0.001/p2<0.001<br>J: p1=0.002/p2=0.009/p3=0.001<br>L: p1<0.001/p2<0.001/p3<0.001
Group B			
ALMA	J: p1=0.001/p2=ns/p3=0.030	ns	F: p1<0.001/p2<0.001/p3<0.001<br>J: p1=0.028/p2=0.003/p3=0.001<br>L: p1=ns/p2=0.011/p3=0.019
Barrett	F: p1=0.044/p2=0.021/p3=0.011<br>J: p1<0.001/p2=0.001/p3=0.001<br>L: p1=0.016/p2=0.008/p3=0.004	ns	F: p1<0.001/p2<0.001/p3<0.001<br>J: p1=0.012/p2=0.006/p3=0.004<br>L: p1<0.001/p2=0.021/p3=ns
Kim	F: p1=ns/p2=0.047/p3=0.011<br>J: p1=0.005/p2=0.003/p3=0.001<br>L: p1=ns/p2=0.021/p3=0.004	ns	F: p1=0.001/p2=0.001/p3=0.001<br>J: p1=ns/p2=0.038/p3=0.037
Latkany	ns	ns	F: p1=ns/p2=ns/p3=0.019
Shammas	F: p1=ns/p2=0.021/p3=0.011<br>J: p1=0.001/p2=0.001/p3=0.001<br>L: p1=ns/p2=0.008/p3=0.004	ns	F: p1<0.001/p2=0.001/p3<0.001<br>J: p1=ns/p2=ns/p3=0.009

Formula: best method compared to the formulas shown in the adjacent columns; A: ALMA Formula; B: Barrett True-K Formula; F: Ferrara Formula, K: Kim Formula, J; Jin Formula; L: Latkany Formula; S: Shammas Formula; p1: level of significance regarding median absolute errors between formulas according to the Friedman test with Bonferroni correction; p2/p3: level of significance regarding the percentages of eyes with a refractive Prediction Error within 0.50D and within 1.00D between formulas, according to Cochran Q Test; ns = p value > 0.05.

Supplementary Table S4. Multiple comparisons among examined formulas according to different Axial Length (AL) and Mean Keratometry (K) Ranges. (Group A and Group B). Only statistically significant differences were reported.

Formula	Group A		Group B	
	K ≤ 36.0D	AL ≤ 26.5mm	K ≤ 36.0D	AL ≤ 26.5mm
ALMA	F: p1=0.002/p2=ns/p3=0.003<br>L: p1<0.001/p2=ns/p3/0.001	F: p1=0.003/p2<0.001/p3=ns<br>J: p1=ns/p2=0.026/p3=ns<br>K: p1=ns/p2=0.08/p3=ns<br>L: p1=0.001/p2/0.001/p3/0.004	F: p1=0.003/p2<0.001/p3=0.002<br>J: p1=ns/p2=0.036/p3=0.028	F: p1=ns/p2=ns/p3=0.010<br>J: p1<0.001/p2=0.031/p3=0.023
Barrett	F: p1<0.001/p2=0.001/p3<0.001<br>J: p1=0.042/p2=0.003/p3=0.013<br>L: p1<0.001/p2=0.001/p3<0.001	F: p1=0.006/p2=0.002/p3=ns<br>L: p1=0.003/p2=0.045/p3=ns	F: P1<0.001/p2=0.001/p3=0.001<br>J: p1=ns/p2=ns/p3=0.012<br>L: P1=ns/p2=ns/p3=0.028	F: p1=0.008/p2=0.014/p3=0.004<br>J: p1<0.001/p2<0.001/p3=0.010<br>L: p1=0.004/p2=ns/p3=ns
Kim	F: P1<0.001/p2=0.001/p3<0.001<br>J: P1=ns/p2=0.003/p3=0.027<br>L: P1<0.001/p2=0.001/p3=0.001	ns	F: P1=0.014/p2=0.003/p3=0.005	F: p1=ns/p2=0.031/p3=0.010<br>J: p1=0.001/p2<0.001/p3=0.023<br>L: p1=ns/p2=0.006/p3=ns
Jin	L: P1=0.042/p2=ns/p3=0.027	L: p1=ns/p2=ns/p3=0.008	ns	
Shammas	F: P1<0.001/p2=0.023/p3<0.001<br>L: P1<0.001/p2=0.023/p3<0.001	F: p1=ns/p2=0.002/p3=ns<br>K: p1=ns/p2=0.045/p3=ns<br>L: p1=ns/p2=0.008/p3=0.008	F: P1=0.003/p2=0.007/p3=0.001<br>J: P1=ns/p2=ns/p3=0.012<br>L: P1=ns/p2=ns/p3=0.028	F: p1=ns/p2=0.031/p2=0.010<br>J: p1<0.001/p2<0.001/p3=0.023<br>L: p1=ns/p2=0.006/p3=ns
	36.0D < K ≤ 38.0D	26.5mm <AL ≤ 28.0mm	36.0D < K ≤ 38.0D	26.5mm <AL ≤ 28.0mm
ALMA	F: P1=0.001/p2=0.003/p3=0.001<br>K: P1=ns/p2=ns/p3=0.049<br>L: P1<0.001/p2=0.003/p3=0.001	F: p1=0.031/p2=0.003/p3<0.001<br>K p1=ns/p2=ns/p3=0.011<br>L: p1<0.001/p2=0.018/p3<0.001	F: P1=ns/p2=ns/p3=0.001	ns
Barrett	F: P1<0.001/p2=0.008/p3<0.001<br>J: P1=ns/p2=ns/p3=0.007<br>K: P1=ns/p2=ns/p3=0.003<br>L: P1<0.001/p2=0.008/p3<0.001	F: p1=0.017/p2=0.003/p3=0.005<br>K: p1=0.045/p2=ns/p3=ns<br>L: p1<0.001/p2=0.018/p3<0.001	F: P1=ns/p2=0.045/p3=0.017	ns
Jin	ns	F: p1=ns/p2=0.008/p3=0.022<br>L: p<0.001/p2=0.039/p3=0.002	ns	ns
Kim	L: P1=0.004/p2=ns/p3=ns	F: p1=ns/p2=0.039/p3=ns	F: P1=ns/p2=0ns045/p3=0.046	ns
Shammas	F: P1<0.001/p2<0.001/p3<0.001<br>J: P1=ns/p2=ns/p3=0.027<br>K: P1=ns/p2=0.040/p3=0.014<br>L: P1<0.001/p2<0.001/p3<0.001	F: p1=ns/p2=0.008/p3=0.022<br>L: p1<0.001/p2=0.039/p3=0.002	F: P1=ns/p2=0.045/p3=0.046	ns
	38.0 < K ≤ 40.0D	28.0mm <AL ≤ 29.5mm	38.0 < K ≤ 40.0D	28.0mm <AL ≤ 29.5mm
ALMA	F: P1=0.001/p2=0.004/p3=0.001<br>K: P1=0.027/p2=ns/p3=0.021<br>L: P1<0.001/p2=0.004/p3<0.001	F: p1<0.001/p2=0.007/p3<0.001<br>J: p1=0.009/p2=0.042/p3<0.001<br>K: p1=0.041/p2=ns/p3=0.002	F: P1=0.009/p2=ns/p3=ns<br>K: P1=0.043/p2=ns/p3=0ns044<br>J: P1<0.001/p2=0.035/p3=0.001<br>L: P1=0.011/p2=ns/p3=0.016	F: p1=0.014/p2=0.004/p3=ns
Barrett	F: P1=0.004/p2=0.018/p3=0.005<br>L: P1<0.001/p2=0.018/p3<0.001	F: p1<0.001/p2=0.007/p3<0.001<br>J: p1=0.007/p2=0.042/p3=0.001<br>K: p1=0.033/p2=ns/p3=0.006<br>L: p1<0.001/p2=0.002/p3<0.001	J: P1=0.014/p2=ns/p3=0.044	F: p1=ns/p2=0.001/p3=ns
Kim	L: p1=ns/p2=ns/p3=0.037	L: p1=0.006/p2=ns/p3=ns	ns	P1=ns/p2=0.033/p3=ns
Jin	F: P1=ns/p2=0.035/p3=ns<br>L: P1<0.001/p2=0.035/p3=0.011	L: p1=0.027/p2=ns/p3=ns	F: P1=ns/p2=ns/p3=0.044	F: p1=ns/p2=0.033/p3=ns
Shammas	F: p1=0.016/p2=0.008/p3=0.005<br>L: p1z0.001/p20.008/p3<0.001	F: p1<0.001/p2=0.002/p3<0.001/<br>J: p1=0.007/p2=0.017/p3<0.001<br>K: p1=0.033/p2=ns/p3=0.006<br>L: p1<0.001/p2=0.001/p3<0.001	ns	F: p1=ns/p2=0.004/p3=ns
	K > 40.0D	AL > 29.5mm	K > 40.0D	AL > 29.5mm
ALMA	F: p1<0.001/p2<0.001/p3=0.004<br>K: p1<0.001/p2<0.001/p3<0.001<br>J: p1=ns/p2=0.002/p3=0.024<br>L: p1<0.001/p2=0.001/p3<0.001	F: p1<0.001/p2=0.011/p3<0.001<br>J: p1=ns/p2=0.030/p3=0.021<br>L: p1<0.001/p2=0.011/p3<0.011	ns	F: p1=0.013/p2=0.011/p3<0.001<br>J: p1=ns/p2=0.026/p3=0.011
Barrett	F: p1=0.002/p2=0.001/p3=ns<br>J: p1=ns/p2=0.024/p3=ns<br>K: p1<0.001/p2=0.002/p3=0.010<br>L: p1=0.001/p2=0.011/p3=0.001	F: p1<0.001/p2=0.004/p3<0.001<br>J: p1=0.011/p2=0.011/p3=0.001<br>L: p1<0.001/p2=0.004/p3<0.001	A: p1=ns/p2=0.031/p3=ns<br>F: p1=0.041/p2=0.012/p3<0.001<br>L: p1=ns/p2=ns/p3=0.019<br>J: p1=ns/p2=ns/p3=0.006	F: p1=0.001/p2=0.026/p3<0.001<br>J: p1=0.017/p2=ns/p3=0.004<br>L: p1=ns/p2=ns/p3=0.026
Jin	L: p1=ns/p2=ns/p3=0.024	ns	ns	ns
Kim	ns	F: p1<0.001/p2=0.030/p3=0.001<br>J: p1=ns/p2=ns/p3=0.043<br>L: p1<0.001/p2=0.030/p3=0.001	F: p1=ns/p2=ns/p3=0.006	F: p1=0.035/p2=ns/p3=0.001<br>J: p1=ns/p2=ns/p3=0.026
Shammas	F: p1=0.032/p2=0.002/p3=ns<br>J: p1=0.048/p2=ns/p3=ns<br>K: p1=0.004/p2=0.005/p3=0.024<br>L: p1=0.011/p2=0.024/p3=0.004	F: p1<0.001/p2=0.004/p3<0.001<br>J: p1=0.014/p2=0.011/p3=0.021<br>L: p1<0.001/p2=0.004/p3<0.001	A: p1=ns/p2=0.031/p3=ns<br>F: p1=ns/p2=0.012/p3=0.002<br>J: p1=ns/p2=ns/p3=0.019<br>K: p1=ns/p2=0.005/p3=0.024	F: p1==.013/p2=ns/p3<0.001<br>J: p1=ns/p2=ns/p3=0.011

Formula: best method compared to the formulas shown in the adjacent columns; A: ALMA Formula; F: Ferrara Formula; K: Kim Formula; J: Jin Formula; L: Latkany Formula; p1: level of significance regarding median absolute errors between formulas according to the Friedman test with Bonferroni correction; p2/p3: level of significance regarding the percentages of eyes with a refractive Prediction Error within 0.50D and within 1.00D between formulas, according to Cochran Q Test; ns= p value > 0.05.
