# Supplementary figures and images for: A No-History Multi-Formula Approach to Improve the IOL Power Calculation after Laser Refractive Surgery: Preliminary Results
Source: J Clin Med. 2023 Apr 15;12(8):2890. doi: 10.3390/jcm12082890 (PMC10144756; doi:10.3390/jcm12082890)

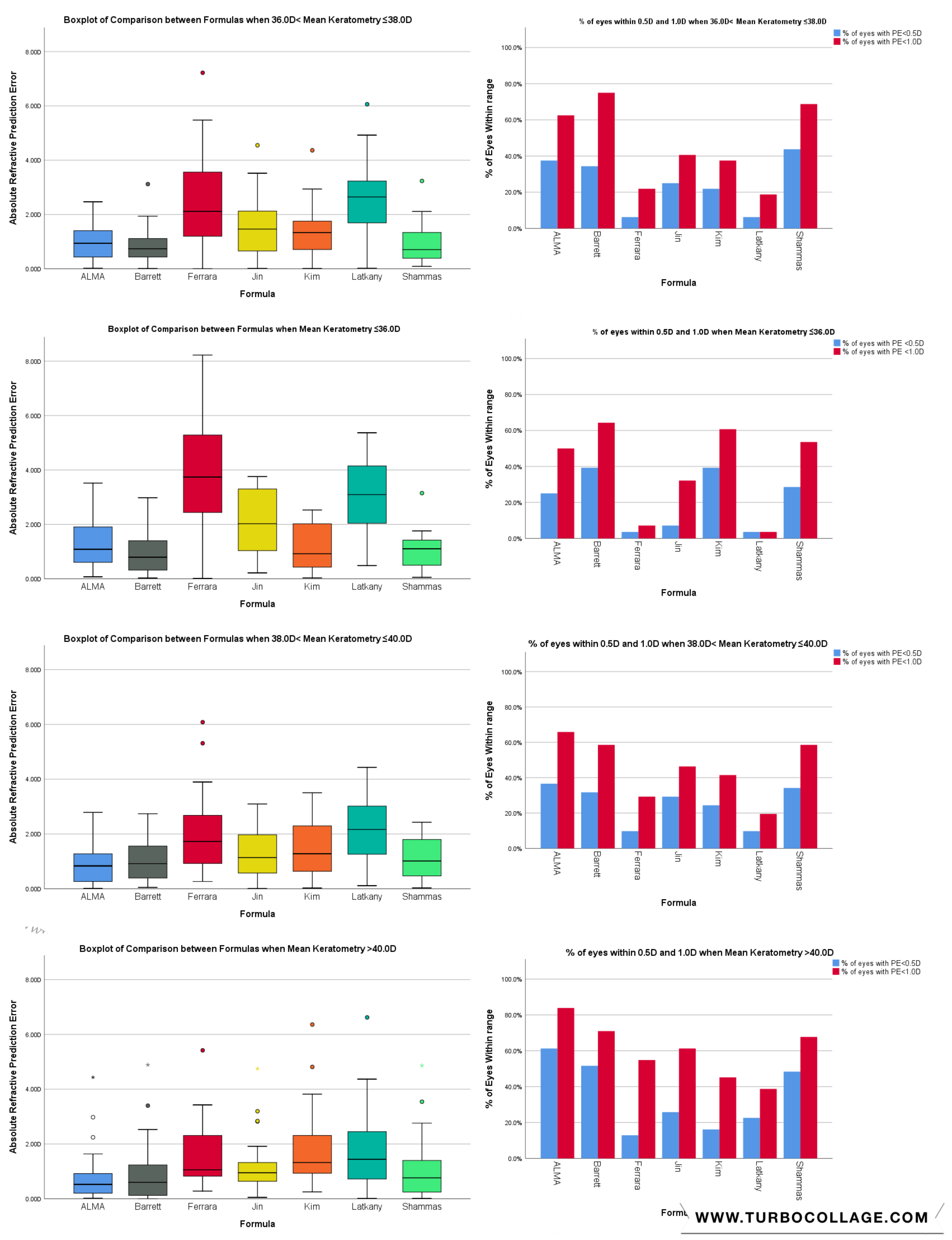

Supplement: Supplementary file 1 [file jcm-12-02890-s001.zip › Figure S1.tiff]

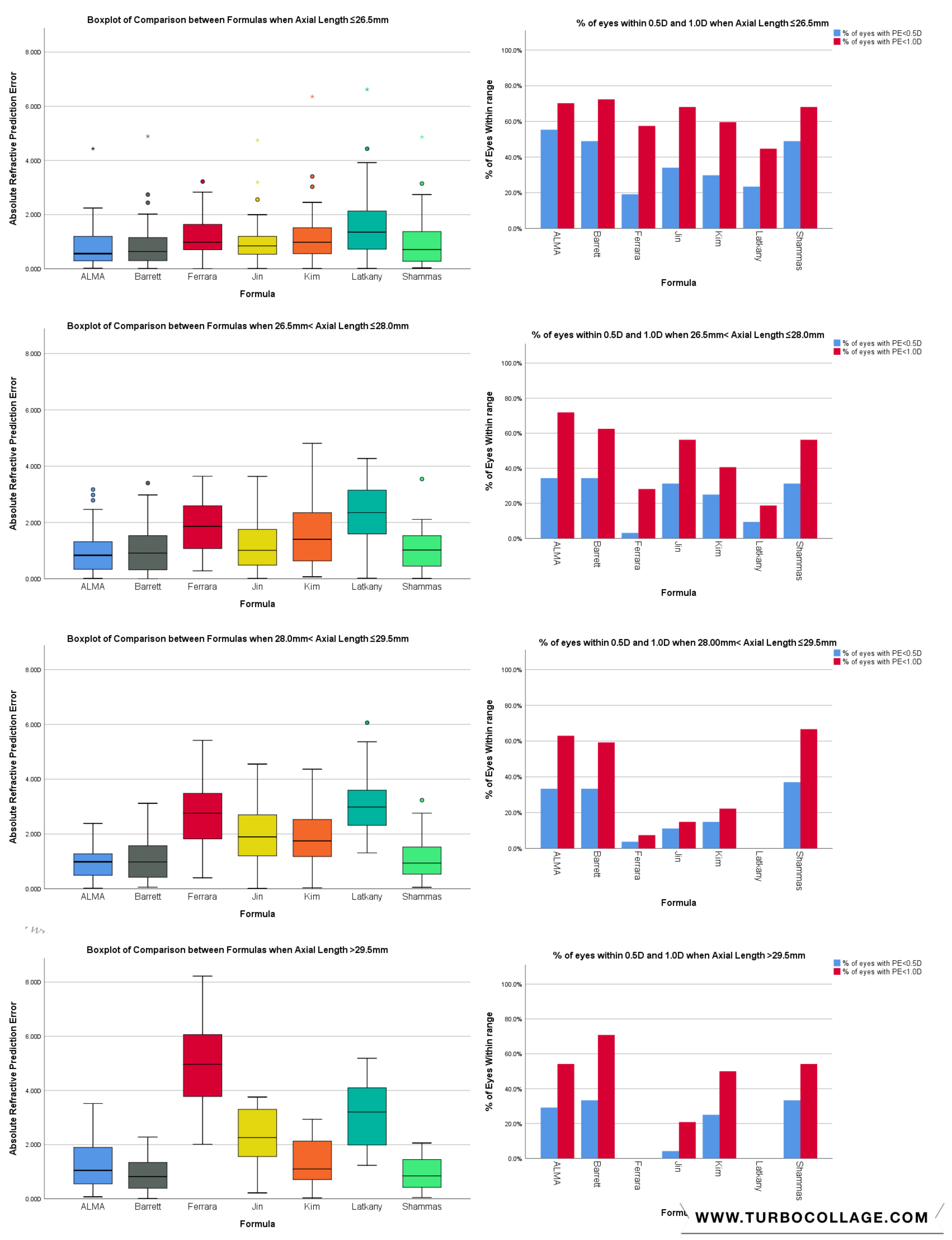

Supplement: Supplementary file 1 [file jcm-12-02890-s001.zip › Figure S2.tiff]

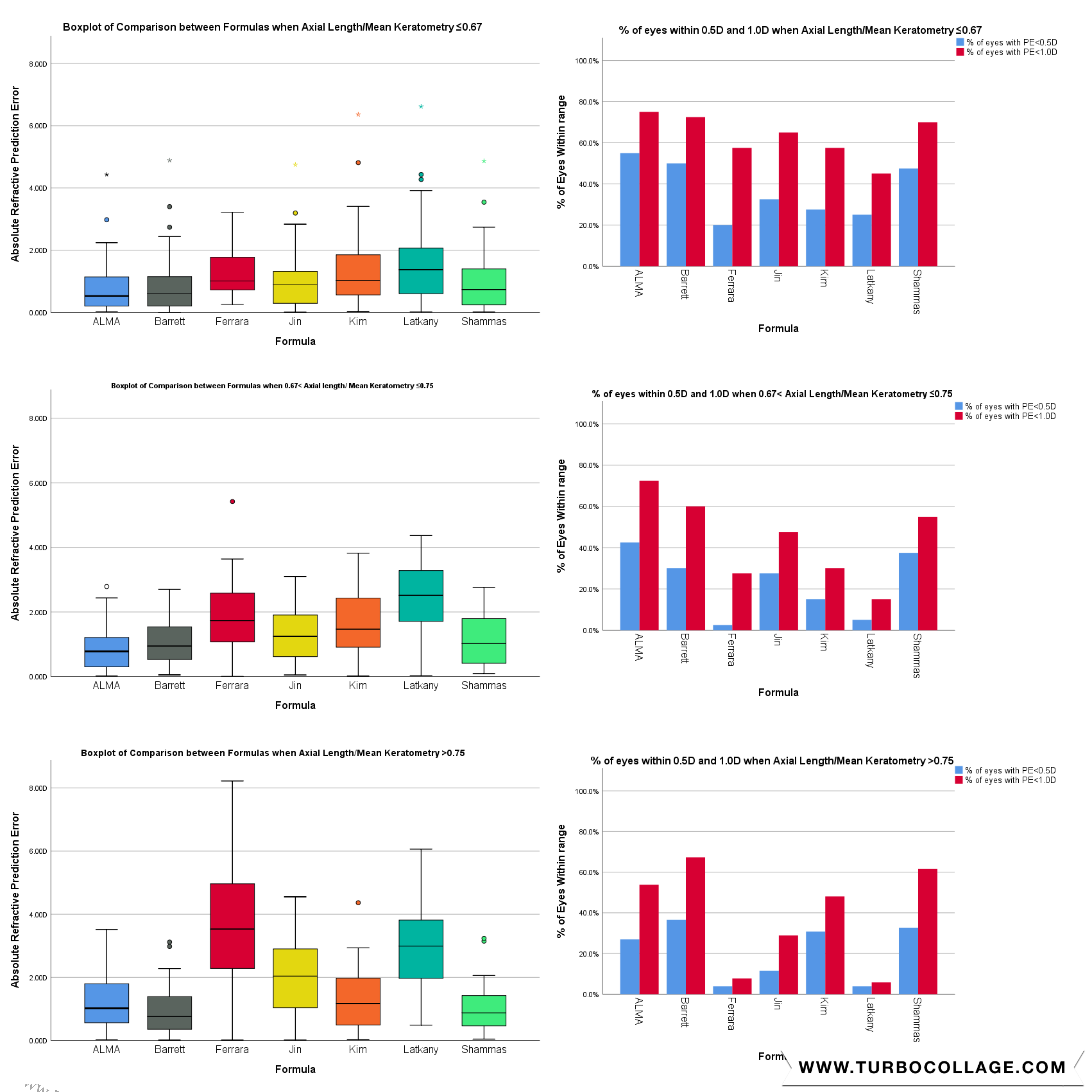

Supplement: Supplementary file 1 [file jcm-12-02890-s001.zip › Figure S3.tiff]

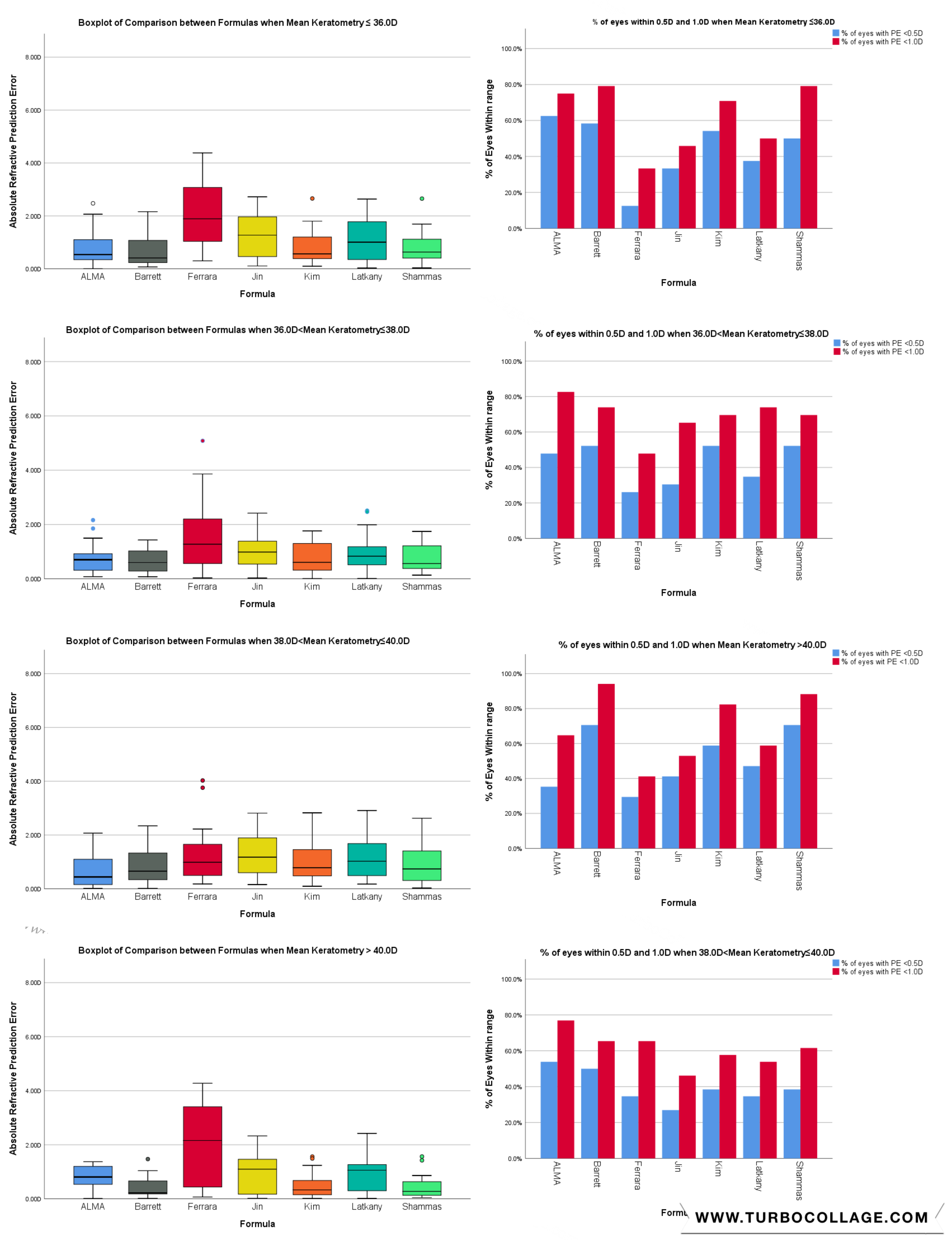

Supplement: Supplementary file 1 [file jcm-12-02890-s001.zip › Figure S4.tiff]

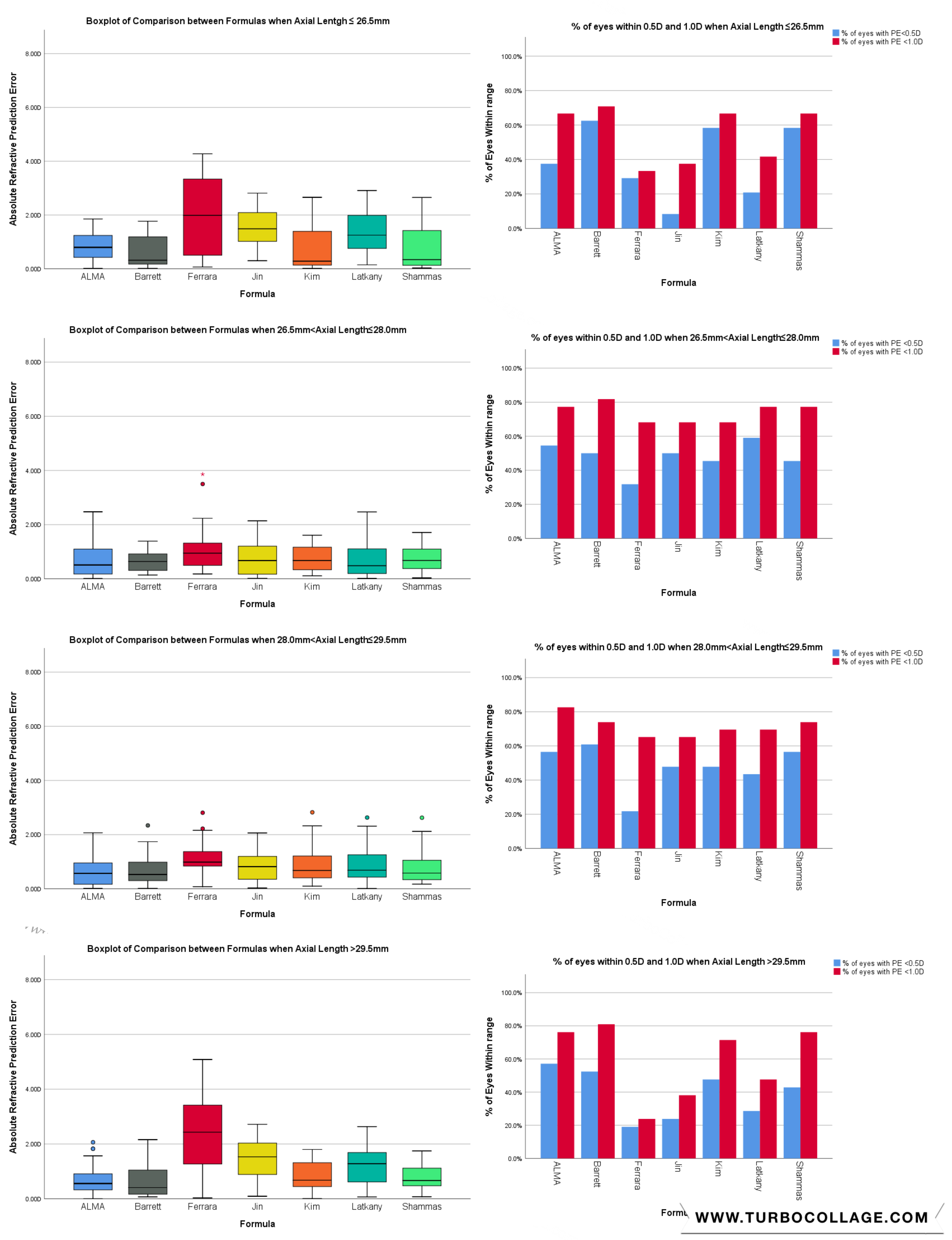

Supplement: Supplementary file 1 [file jcm-12-02890-s001.zip › Figure S5.tiff]

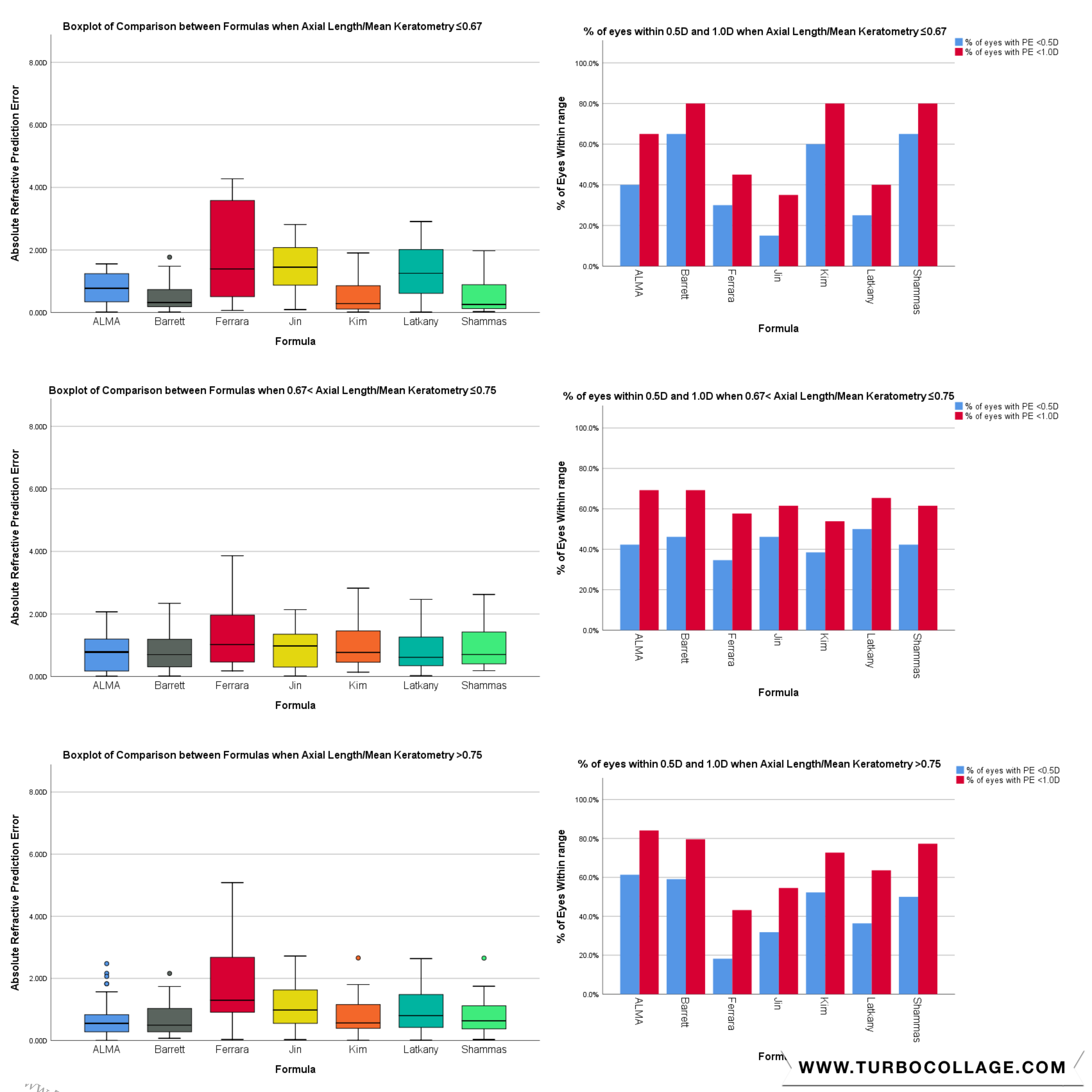

Supplement: Supplementary file 1 [file jcm-12-02890-s001.zip › Figure S6.tiff]
